# Endogenous RGS14 is a cytoplasmic-nuclear shuttling protein that localizes to juxtanuclear membranes and chromatin-rich regions of the nucleus

**DOI:** 10.1371/journal.pone.0184497

**Published:** 2017-09-21

**Authors:** Mary Rose Branch, John R. Hepler

**Affiliations:** Department of Pharmacology, Emory University School of Medicine, Atlanta, Georgia, United States of America; University of North Dakota, UNITED STATES

## Abstract

*R*egulator of *G* protein *s*ignaling 14 (RGS14) is a multifunctional scaffolding protein that integrates G protein and H-Ras/MAPkinase signaling pathways to regulate synaptic plasticity important for hippocampal learning and memory. However, to date, little is known about the subcellular distribution and roles of endogenous RGS14 in a neuronal cell line. Most of what is known about RGS14 cellular behavior is based on studies of tagged, recombinant RGS14 ectopically overexpressed in unnatural host cells. Here, we report for the first time a comprehensive assessment of the subcellular distribution and dynamic localization of endogenous RGS14 in rat B35 neuroblastoma cells. Using confocal imaging and 3D-structured illumination microscopy, we find that endogenous RGS14 localizes to subcellular compartments not previously recognized in studies of recombinant RGS14. RGS14 localization was observed most notably at juxtanuclear membranes encircling the nucleus, at nuclear pore complexes (NPC) on both sides of the nuclear envelope and within intranuclear membrane channels, and within both chromatin-poor and chromatin-rich regions of the nucleus in a cell cycle-dependent manner. In addition, a subset of nuclear RGS14 localized adjacent to active RNA polymerase II. Endogenous RGS14 was absent from the plasma membrane in resting cells; however, the protein could be trafficked to the plasma membrane from juxtanuclear membranes in endosomes derived from ER/Golgi, following constitutive activation of endogenous RGS14 G protein binding partners using AlF_4_¯. Finally, our findings show that endogenous RGS14 behaves as a cytoplasmic-nuclear shuttling protein confirming what has been shown previously for recombinant RGS14. Taken together, the findings highlight possible cellular roles for RGS14 not previously recognized that are distinct from the regulation of conventional GPCR-G protein signaling, in particular undefined roles for RGS14 in the nucleus.

## Introduction

Heterotrimeric G proteins couple to cell surface receptors to transduce signals by hormones and neurotransmitter across the plasma membrane and into the interior of cells to mediate all aspects of cell and organ physiology [1,2]. The family of RGS proteins bind directly to G proteins and their receptors to serve as GTPase activating proteins (GAPs) and negatively regulate G protein signaling events [3]. Whereas most RGS proteins are relatively simple proteins that act as dedicated GAPs activated Gα subunits, others are more complex with various domains for binding partners that mediate unconventional G protein signaling events. One such RGS protein is RGS14, a multifunctional scaffolding protein that interacts with specific G protein alpha subunits (Gαi/o) and activated H-Ras to integrate G protein and MAP kinase signaling pathways [4–9]. Our previous work has shown that RGS14 is highly enriched within pyramidal neurons of the hippocampus where it serves as an important natural suppressor of long-term potentiation (LTP) and hippocampal-dependent learning and memory (10). Due to the key role that RGS14 serves in regulating synaptic plasticity, we felt it to be critically important that we determine where endogenous RGS14 operates within a neuronal cell line for a better understanding of its cellular roles and functions and the possible mechanism(s) by which it limits LTP.

To date, most of what is known and assumed regarding the cellular functions of RGS14 is based on the behavior and subcellular localization of tagged (e.g. GFP or FLAG) and overexpressed recombinant RGS14 introduced into unnatural host cells (e.g. HEK or HeLa cells). By contrast, almost nothing is known about the subcellular distribution of endogenous RGS14 in neuronal cells. Efforts to study this have been hampered by the restriction of RGS14 protein expression to CA2 hippocampal neurons of adult mice [10,11]. Neuron cultures from adult mice are not possible; therefore, prior study of RGS14 behavior in cells has been limited to that of ectopically expressed recombinant RGS14 in unnatural host cells [7,8,12–14]. In those studies, recombinant epitope-tagged RGS14 localizes predominantly within the cytosol, and can be recruited to the plasma membrane by co-expression with its Gαi/o binding partners or trapped in the nucleus following pharmacological blockade of nuclear export. Whether this protein behavior accurately reflects the subcellular distribution and behavior of endogenous RGS14 in a neuronal cell line is unknown. Since mislocalization of exogenously expressed proteins can give a false impression of the endogenous protein's distribution and *in vivo* functionalities, the goal of this study was to investigate the subcellular localization of endogenous RGS14 in its native cellular environment.

We previously reported that rat B35 neuroblastoma cells naturally express endogenous RGS14 by immunoblot analysis [15]. In this study, we took advantage of B35 cells to examine and determine the subcellular distribution and dynamic localization of endogenous RGS14 in a neuronal cell line. We find that the endogenous RGS14 protein exhibits a different pattern of subcellular localization and distribution than has been reported for recombinant protein, suggesting novel roles for RGS14 not previously considered. Our findings suggest that endogenous RGS14 may not serve canonical GPCR-G protein signaling roles at the plasma membrane like other RGS proteins but, rather, it may serve distinct non-canonical roles within the nucleus, possibly regulating gene expression.

## Materials and methods

### Plasmids and antibodies

The FLAG-RGS14 and eGFP-RGS14 cDNA used in this study were generated as described previously [13] using rat RGS14 cDNA (Genbank accession number U92279) [6]. For a comprehensive list of antibodies and antibody concentrations used, see Table 1.

**Table 1:** List of antibodies used in this study.

**Primary antibody**	**Host**	**Provider**	**Application**
RGS14 pAb	rabbit	Proteintech	ICC (1:450); IB (1:500)
FLAG	rabbit	Sigma	ICC (1:1000)
Lamin A/C	mouse	Cell Signaling	ICC (1:3000); IB (1:3000)
OPA1	mouse	Biosciences BD	IB (1:1000)
GAPDH	mouse	Santa Cruz	IB (1:5000)
414 mAb (NPC)	mouse	A kind gift from Dr. Maureen Powers, Emory University	ICC (1:8000)
KDEL receptor (KDELR) ^§^	mouse	Stressgen	ICC (1:1000)
RNA polymerase II Ser2P (H5)	mouse	A kind gift from Dr. William G. Kelly, Emory University	ICC (1:1000)
HSP60	mouse	Enzo Life Sciences	ICC (1:5000)
GM130 ^§^	mouse	BD Transduction	ICC (1:1000)
α-tubulin ^§^	mouse	Sigma	ICC (1:2000)
γ-tubulin ^§^	mouse	Sigma	ICC (1:2000)
Mannose 6 phosphate receptor: CI/300 ^§^	mouse	Gift to the Kahn lab from Annette Hille-Rehfeld	ICC (1:1000)
**Secondary antibody**	**Host**	**Provider**	**Application**
Anti-mouse Alexa 488	goat	Molecular Probes	ICC (1:1000)
Anti-rabbit Alexa 594	goat	Molecular Probes	ICC (1:1000)
Anti-rabbit HRP-conjugated IgG	goat	BioRad	IB (1:25,000)
Anti-mouse HRP-conjugated IgG	goat	Rockland Immunochemicals	IB (5000)

ICC: Immunocytochemistry; IB: Immunoblotting

^§^ Antibodies generously provided by Dr. Richard Kahn’s lab, Emory University

### Cell culture and transfections

Rat neuroblastoma (B35), Human cervical carcinoma (HeLa), African Green Monkey kidney (Cos7), human glioblastoma (SF295), and human embryonic kidney (HEK293) cells were all maintained in 1X Dulbecco’s modified eagle medium (DMEM) with phenol red indicator (Mediatech) supplemented with 10% fetal bovine serum (FBS) (Atlanta Biologicals, 5% after transfection), 100 U/mL penicillin (Mediatech), and 100 mg/mL streptomycin (Mediatech) in a humidified environment at 37°C with 5% CO_2_. For immunofluorescence experiments, cells were seeded onto poly-D-lysine-coated glass coverslips. All transient transfections were performed using polyethyleneimine (PEI; Polysciences, Inc.) as previously described [16].

### Leptomycin B treatment

Leptomycin B (LMB; Santa Cruz), a CRM1-dependent nuclear export inhibitor [17], was diluted in 70% ethanol. Treatment of B35 cells with LMB was as previously described (Shu et al., 2007). Where indicated, LMB (or ethanol control) was added to the culture medium at a final concentration of 20 nM and cells were incubated at 37°C for the indicated amounts of time up to 3 hours, followed by fixation and subsequent immunofluorescence staining.

### Cell cycle synchronization

To induce G1 cell cycle arrest, B35 cells were plated onto coverslips in complete DMEM media containing 10% FBS, 100 U/mL penicillin, and 100 mg/mL streptomycin. After 24 hours, complete media was replaced with serum-free media (DMEM without FBS) for 24 hours. To synchronize cells in S or G2, a double thymidine block and release method was used [18]. Thymidine (Sigma) was added to cells at a final concentration of 2 mM for 19 hours to arrest cells at G1/S. Cells were washed in 1X PBS and incubated in fresh media for 8 hours followed by a second treatment with 2 mM of thymidine for an additional 15 hours. At the final release, cells were washed in 1X PBS and incubated in fresh media. B35 cells were then fixed at various time points following thymidine release and processed for immunocytochemistry. Cell cycle stages were confirmed by immunostaining for gamma-tubulin to assess centrosome duplication and positioning.

### Subcellular fractionation

B35 cells were lysed and fractioned to isolate intact nuclei and cytosol in a protocol modified from [19]. B35 cells were washed and collected in ice cold 1X PBS by centrifuging at 1000 g at 4°C for 5 min. Cells were then resuspended in 10 volumes of Nonidet-P40 lysis buffer (10 mM HEPES, pH 7.5; 10 mM KCl; 0.1 mM EDTA; 1 mM dithiothreitol (DTT); 0.5% Nonidet‐40; protease inhibitor cocktail (Roche)) and allowed to swell in ice for 12 min with intermittent mixing. Samples were then vortexed at max speed for 10–12 sec to disrupt cell membranes, and 10% of the volume was removed for later assessing whole cell lysates by immunoblotting. After centrifugation at 1,200 g for 8 min, the supernatant was collected as cytoplasmic extract and supplemented with 240 mM NaCl. The remaining pellet was washed twice in lysis buffer then re-suspended in nuclear extraction buffer (20 mM HEPES, pH 7.5; 400 mM NaCl; 1 mM EDTA; 1 mM DTT; protease inhibitor cocktail), allowed to swell on ice for 30 min, and centrifuged at 12,000 g for 15 min. Resulting supernatant was used as the nuclear extract. 5% of each sample was removed to use for the input prior to immunoprecipitation.

### Immunoprecipitation

Cytoplasmic and nuclear extracts were used to immunoprecipitate RGS14 using standard protocols. Briefly, extracts were incubated with a 1:100 dilution of RGS14 polyclonal antibody (Proteintech) overnight at 4°C. Beads only control samples were also incubated overnight at 4°C without the addition of antibody. 50 μl of protein A-Sepharose beads were washed and blocked in 3% BSA for 1 hour at 4°C, and incubated with samples for an additional 1.5 hours at 4°C. Beads were then washed 4 times with 0.1% Tween-20 in 1X PBS, re-suspended in 2X Laemmli sample buffer, and heated at 95°C for 5 min.

### Immunoblotting

Mouse brain lysates were kindly provided by Paul Evans (Hepler lab, Emory University) and prepared as in (11). Immunoblotting experiments were carried out as descried in (11) with a few modifications. Briefly, B35 cells were lysed on ice in buffer containing 50 mM Tris-HCl, pH 8.0, 150 mM NaCl, 1 mM EGTA, 1 mM EDTA,2 mM dithiothreitol, 10 mM MgCl_2_, protease inhibitor cocktail (Roche), and 1% TritonX-100. Cell lyastes were incubated on a rotator for 1 hour at 4°C, and then cleared by centrifugation at 100,000 × g for 30 min at 4°C. Lysates were mixed with Laemmli sample buffer and boiled for 5 min at 95°C. Samples from the cell lysates and mouse brain homogenates were loaded onto 11% acrylamide gels, resolved by SDS-PAGE, transferred to nitrocellulose membranes. After blocking nitrocellulose membranes for 1 hour at room temperature in blocking buffer containing 5% nonfat milk (w/v), 0.1% Tween-20, and 0.02% sodium azide, diluted in 20 mM Tris buffered saline, pH 7.6, membranes were incubated with primary antibodies diluted in the same buffer overnight at 4°C or for 2 hours at room temperature. Membranes were then washed in Tris buffered saline containing 0.1% Tween-20 (TBST) and incubated with either an anti-mouse (1:5000) or anti-rabbit (1:25,000) HRP-conjugated secondary antibody diluted in TBST for 1hour at room temperature. Protein bands were detected by enhanced chemiluminescence.

### Immunofluorescence

In this study, we extensively compared native RGS14 staining following various fixation and permeabilization methods. To visualize native RGS14, unless otherwise stated, B35 cells seeded onto PDL-coated coverslips were fixed in 3.7% paraformaldehyde (PFA) in PHEM buffer (60 mM PIPES, 25 mM HEPES, 10 mM EGTA, 2 mM MgCl_2_, pH 6.9. Tris-Glycine; 200 mM, Tris, 0.75% glycine, pH 7.4) for 10 minutes at room temperature (RT), followed by permeabilization with 100% ice-cold methanol for 5 min at -20°C. Coverslips were then rinsed three times in PBS-Tween (0.05% Tween in 1X PBS), blocked in PBS containing 8% BSA for 1 hour at room temperature, and incubated with primary antibody in PBS containing 4% BSA (antibody buffer) overnight at 4°C. Coverslips were then washed for 5 min in PBS-Tween three times, incubated with Alexa 594 goat anti-rabbit and/or Alexa 488 goat anti-mouse secondary antibody (1:1000; Molecular Probes) in antibody buffer for 1–1.5 hours at room temperature, washed for 5 min in PBS-Tween two times, counter-stained with Hoechst 33342 to visualize DNA, washed again in PBS-Tween, and mounted onto slides with ProLong Diamond Antifade mounting media (Invitrogen). All cells transfected with FLAG-RGS14 and B35 cells co-stained with Rhodamine Phalloidin (F-actin) or anti-HSP60 (mitochondria) were fixed as above and permeabilized with 0.1% Triton-X in PBS for 10 min at RT. Though native RGS14 distribution looked similar with all fixation/permeabilization methods, with the exception of saponin permeabilization, which does not permeabilize the nuclear membrane, we found that PFA/PHEM fixation and methanol permeabilization resulted in optimal staining.

### Antibody pre-adsorption

In experiments testing antibody specificity, diluted anti-RGS14 polyclonal antibody was incubated with 5X (μg/mL) excess of purified rat RGS14 [14] on a rotator overnight at 4°C prior to immunoblot or immunofluorescence. Immunoblots probed with antibody alone and with antibody pre-absorbed with purified protein were processed in parallel.

### Confocal microscopy

Confocal imaging was performed using a 60X oil immersion objective on Olympus FV1000. Fluorescence channels were scanned sequentially and averaged to avoid bleed through. For immunofluorescence pre-adsorption experiments comparing staining with RGS14 polyclonal antibody, pre-adsorbed antibody, and secondary only, all images were acquired and processed using identical confocal settings (exposure time, gain, intensity). Images were processed and intensity graphs were generated using ImageJ software (http://rsb.info.nih.gov/ij/).

### 3D-structured illumination microscopy (SIM)

Super-Resolution 3D-SIM images were acquired with a DeltaVision OMX Blaze system (GE Healthcare) equipped with a 60X/1.42 NA oil immersion objective, 405, 488, 568, and 642 nm diode lasers, and sCMOS cameras. Hoechst, Alexa 488, and Alexa 594 staining were excited with 405, 488, and 568 nm diode lasers, respectively. To limit spectral cross-talk, SIM data were acquired in alternating excitation mode. Image stacks were acquired with a z-distance of 0.125 μm and computationally reconstructed to generate super-resolution optical serial sections. Color alignment and SI reconstruction were performed with Softwork software package version 6.5.2 (GE Healthcare). Subsequent image processing was performed using ImageJ software.

### Activation of B35 cell endogenous G protein with AlF_4_¯

For aluminum tetrafluoride (AlF_4_¯)- induced G protein activation, B35 cells were incubated with Tyrode’s solution (140 mM NaCl, 5 mM KCl, 1 mM Mg Cl_2_, 1 mM CaCl_2_, 0.37 mM NaH_2_PO_4_, 24 mM NaHCO_3_, 10 mM HEPES, and 0.1% glucose, pH 7.4) supplemented with or without (control) 10 mM NaF, 9 mM MgCl_2_, and 30 μM AlCl_3_ for indicated times at 37°C.

### Analysis of RGS14 translocation in B35 cells

Translocation of endogenous RGS14 in response to AlF_4_¯-induced G protein activation was assessed by immunofluorescence and confocal microscopy. After staining B35 cells treated with AlF_4_¯for 10 min (n = 35) or left untreated (control; n = 35) with anti-RGS14 polyclonal antibody to detect endogenous RGS14, the fluorescence intensity around the RGS14- enriched juxtanuclear membrane was compared to the fluorescence intensity of RGS14 within the cytoplasm using ImageJ software. Hoechst DNA stain, visualized under the DAPI channel on the confocal microscope, was used to locate the nucleus, and the area around the nuclear membrane was traced with the freehand tracing tool in ImageJ. Translocation was considered a significant difference in relative fluorescence staining around the nuclear membrane between untreated (control) and AlF_4_¯- treated cells. Relative nuclear membrane fluorescence was determined by dividing the mean fluorescence intensity (total fluorescence/area) around the juxtanuclear membrane by the mean fluorescence intensity in a comparable sized area within the cytosol. All non-dividing cells from randomly selected fields pooled from three independent experiments were included in the analysis. Statistical analysis was carried out using GraphPad Prism software. Comparisons between control and AlF_4_¯ -treated B35 cells (n = 35 cells per group) were performed using a two-tailed unpaired t-test with *P* < 0.05 considered statistically significant. Data are reported as mean +/- s.d.

## Results

### Endogenous RGS14 is expressed in B35 neuroblastoma cells

**Fig 1:**
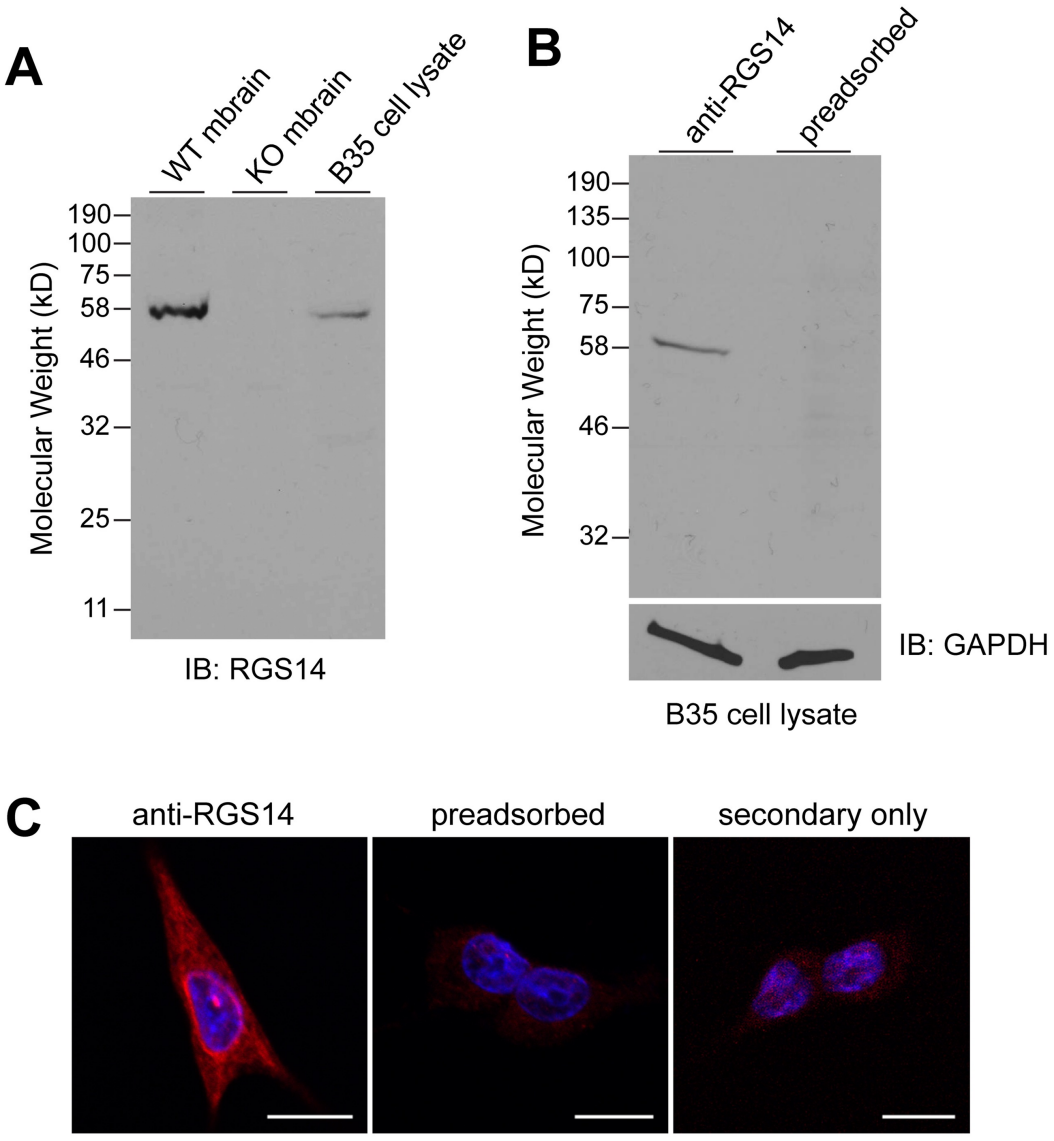
RGS14 polyclonal antibody specifically recognizes endogenous RGS14 in mouse brain and B35 neuroblastoma cells. (A) The presence of endogenous RGS14 in brain from wild type (WT) and RGS14 knockout (KO) mice, and B35 rat neuroblastoma cells was analyzed by SDS-PAGE and immunoblotting with an RGS14 polyclonal antibody. (B) Equivalent amounts of B35 cell lysate were resolved by SDS–PAGE and transferred to nitrocellulose membranes. Membranes were probed with an RGS14 antibody or RGS14 antibody pre-adsorbed with five-fold excess (ng protein) purified full-length rat RGS14. (C) Confocal images of B35 cells immunostained with an RGS14 antibody, RGS14 antibody pre-adsorbed with five-fold excess purified full-length RGS14, or no primary antibody (*secondary only*) followed by Alexa 594 secondary antibody (*red*). Nuclei were counterstained with Hoechst (*blue*). Scale bar, 10 μm. All images were acquired and processed using identical settings. Cells shown are representative of approximately 600 cells observed from 40 fields of view across three independent experiments.

We previously reported that rat B35 neuroblastoma cells naturally express RGS14 protein at detectable levels [15]. We used these cells to determine the subcellular localization and cellular distribution of endogenous RGS14. RGS14 encodes a predicted 61 kDa protein in mouse and rat. Using a novel and previously uncharacterized polyclonal anti-RGS14 antibody (Proteintech), we observed that the antibody recognizes RGS14 in both mouse brain and rat B35 cells, consistent with our previous observations [11,15]. The RGS14 antibody detected a single band at approximately 60 kD in wild type mouse brain lysates and B35 cell lysate, but not in lysates from RGS14 knockout (KO) mouse brain (Fig 1A)) and showed a similar staining pattern in wild type mouse brain sections as the validated monoclonal antibody (10,20), indicating that the antibody specifically recognizes endogenous RGS14. To further validate the antibody specificity, we found that pre-adsorption of the antibody with purified recombinant rat RGS14 completely blocked staining of the 60 kDa band (Fig 1B). We next determined if the antibody recognized RGS14 in fixed B35 cells detected by immunocytochemistry (Fig 1C). The antibody recognized diffuse cellular staining that is not due to secondary antibody, and is completely blocked by preadsorption of the antibody with purified rat RGS14 protein. We further found that the RGS14 antibody recognizes overexpressed GFP-RGS14 in transiently transfected B35 cells, as antibody staining completely colocalized with intrinsic GFP fluorescence (S1 Fig). Taken together, these findings demonstrate that the polyclonal RGS14 antibody specifically recognizes endogenous RGS14 expressed in rat B35 neuroblastoma cells and validate its use in both western blot and immunofluorescence experiments.

### Endogenous RGS14 localizes to various subcellular compartments in B35 cells

**Fig 2:**
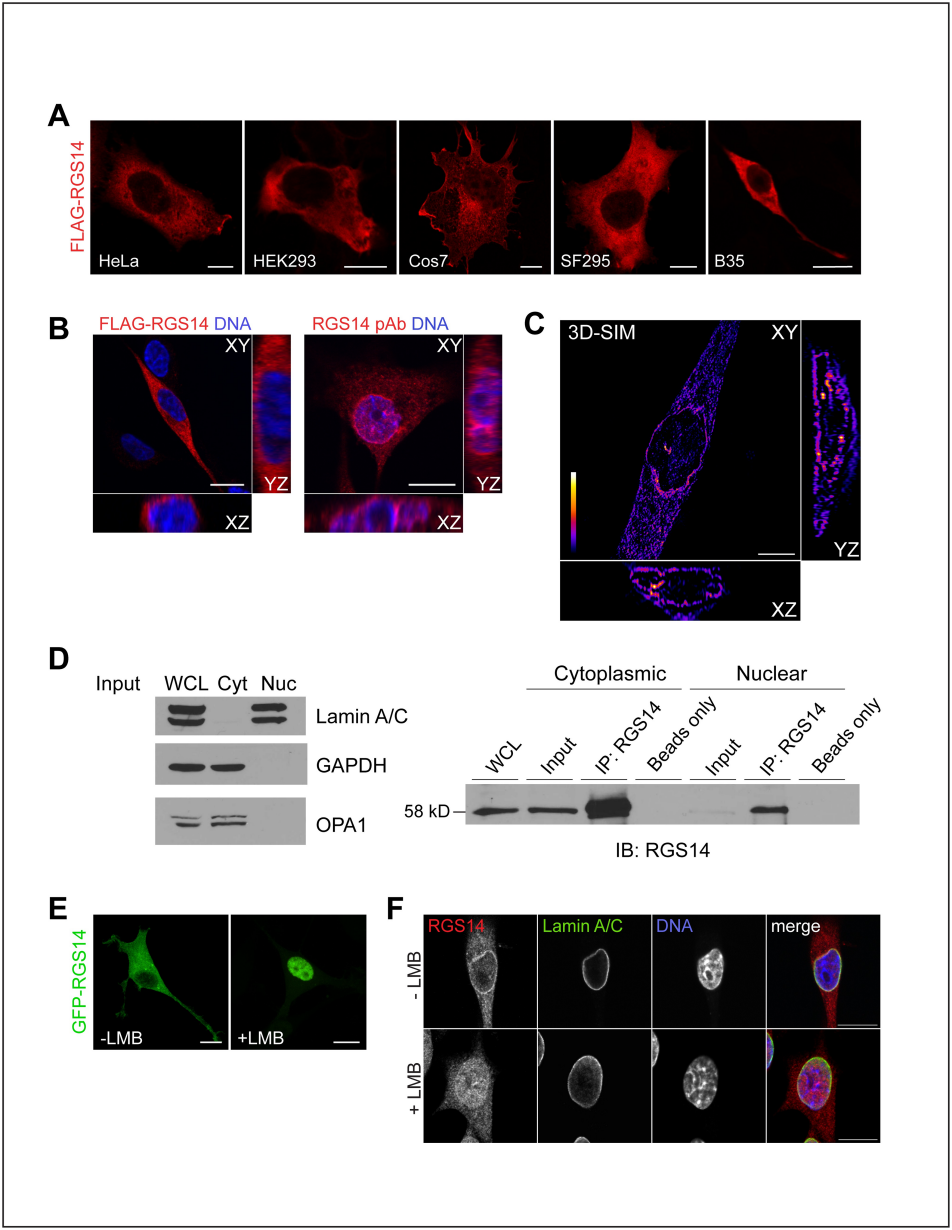
Endogenous RGS14 is enriched at juxtanuclear membranes and shuttles between the cytoplasm and nucleus in B35 cells. (A) FLAG-RGS14 predominantly localizes to the cytoplasm under basal conditions when expressed in various cell lines. HeLa, HEK293, Cos7, SF295, and B35 cells were transfected with 250–500 ng of FLAG-RGS14 cDNA, immunostained with a FLAG antibody and visualized by confocal microscopy. Scale bar, 10 μm. (B) Optical mid-section (XY) and orthogonal views (XZ, YZ) of representative confocal z stacks of B35 cells expressing exogenous FLAG-RGS14 immunostained with a FLAG antibody (*left*) or endogenous RGS14 immunostained with an RGS14 polyclonal antibody (RGS14 pAb, *right*). DNA was visualized with Hoechst counterstain (*blue*). While exogenously expressed FLAG-RGS14 localizes to the cytoplasm, endogenous RGS14 appears to localize to various subcellular compartments, including cytoplasmic puncta, nuclear periphery, nuclear matrix, and nuclear foci. (C) Optical mid-section (XY) and orthogonal views (XZ, YZ) of a z-stack acquired using 3D-Structured Illumination Microscopy (3D-SIM) of a B35 cell immunostained with RGS14 pAb. Image shows a heat map of pixel intensity (‘Fire’ LUT, ImageJ). (D) Cellular fractionation of B35 cells into cytoplasmic and nuclear fractions. To assess fractionation purity (*Left*), whole cell lysate (WCL) and equal volumes of cytoplasmic (Cyt) and nuclear (Nuc) fractions were probed with antibodies against Lamin A/C (nuclear membrane), GAPDH (soluble cytosol), and OPA1 (mitochondria). Immunoprecipitation of endogenous RGS14 from both cytoplasmic and nuclear fractions (*Right*) confirms native RGS14 localizes to both the cytoplasm and nucleus. Cytoplasmic and nuclear fractions were prepared using equivalent volumes of buffer and used for immunoprecipitation (IP) of RGS14. Input represents 10% of IP sample volume. Both exogenous GFP-RGS14 (E) and endogenous RGS14 (F) accumulate in the nucleus in the presence of the inhibitor of CRM1-dependent nuclear export, leptomycin B (LMB). Untransfected B35 cells or cells transfected with GFP-RGS14 were incubated with 20 nM of LMB or ethanol control for 3 hours. (E) Representative maximum intensity projections of confocal z-stacks of B35 cells expressing GFP-RGS14 incubated with ethanol control (-LMB) or 20 nM of LMB (+LMB) for 3 hours. (F) Representative confocal image (optical mid section) of an untransfected B35 cells immunostained with RGS14 pAb (*red*) and anti-Lamin A/C (*green*) to outline the nuclear membrane, and counterstained with Hoechst (*blue*) to visualize the nucleus.. Cells shown are representative of approximately 600 cells observed from 40 fields of view across 3 independent experiments.

We next compared the subcellular distribution of endogenous RGS14 in B35 cells with that of recombinant FLAG-tagged RGS14 (FLAG-RGS14) in various non-host cells (Fig 2A). In line with findings from our previous studies [4,5,14,20], ectopically expressed FLAG- RGS14 detected by immunofluorescence and confocal microscopy was found to localize predominantly in the cytosol when over-expressed in various cell lines, including B35 cells (Fig 2A).

To further clarify the subcellular localization of endogenous RGS14 in B35 cells, we first compared several cell fixation protocols for immunofluorescence to determine optical fixation and permeabilization conditions. While the subcellular distribution of endogenous RGS14 appeared similar with most fixation and permeabilization protocols tested (S2 Fig), we observed optimal detection of endogenous RGS14 when B35 cells were fixed with 3.7% paraformaldehyde in PHEM (cytoskeletal stabilizing buffer) at room temperature and permeabilization with ice cold methanol at -20°C (see Materials and methods section). Under these conditions, immunolabeled endogenous RGS14 in B35 cells localized within cytoplasmic puncta, in unidentified nuclear bodies, and at the nuclear periphery (Fig 2B), a staining pattern that differed from the diffuse cytoplasmic localization of ectopically expressed recombinant FLAG-RGS14 (Fig 2A). Notably, the robust RGS14 signal around the nuclear periphery was diminished in most cells permeabilized with saponin (S2 Fig), which does not permeabilize the nuclear membrane [21], suggesting that a subpopulation of endogenous RGS14 resides on the nuclear side of the nuclear envelope. Using 3D-structured illumination microscopy (3D-SIM) [22] to examine the localization of endogenous RGS14 at the super-resolution level, we found RGS14 staining appeared in a discontinuous punctate pattern along the nuclear periphery and also in discrete foci within the nucleus (Fig 2C). To further confirm the nuclear localization of RGS14, we attempted to immunoprecipitate endogenous RGS14 from cytoplasmic and nuclear B35 cell fractions (Fig 2D). Immunodetection of the cytosolic marker GAPDH, mitochondria marker OPA1, and nuclear membrane marker Lamin A/C confirmed the purity of the cytoplasmic and nuclear inputs. Immunoprecipitation of RGS14 from these cell fractions confirmed the presence of endogenous RGS14 in both B35 cell cytoplasmic and nuclear fractions (Fig 2D). Using this approach, we determined that endogenous RGS14 localizes to both the cytoplasm and, to a lesser extent, the nucleus under basal conditions in B35 cells.

We next examined whether endogenous RGS14 is a cytoplasmic-nuclear shuttling protein. We and others previously reported that RGS14 contains both a nuclear localization sequence (NLS) and a nuclear export sequence (NES), and that recombinant RGS14 accumulates in the nucleus following treatment of cells with Crm1-dependent nuclear export inhibitor, leptomycin B (LMB) [12,13]. In agreement with these previous reports of recombinant RGS14 behavior in HeLa cells, we found that GFP-RGS14 exhibits a predominantly cytoplasmic localization in B35 cells under basal conditions, but accumulates in the nucleus following treatment of cells with LMB (Fig 2E, S3 Fig). We next sought to determine whether endogenous RGS14 also shuttles between the cytoplasm and nucleus in a Crm1-dependent manner. After treatment with LMB (final concentration, 20 ng/mL for 3 h), we observed a significant increase in the localization of endogenous RGS14 to the nuclear matrix and a concomitant decrease in localization around the nuclear periphery (Fig 2F), suggesting that endogenous RGS14 is continuously shuttling between the nucleus and cytoplasm, which may explain its prominent localization at the nuclear periphery.

### Endogenous RGS14 localizes as diffuse puncta in the cytoplasm and is highly enriched in the juxtanuclear membranes adjacent to endoplamsmic reticulum

**Fig 3:**
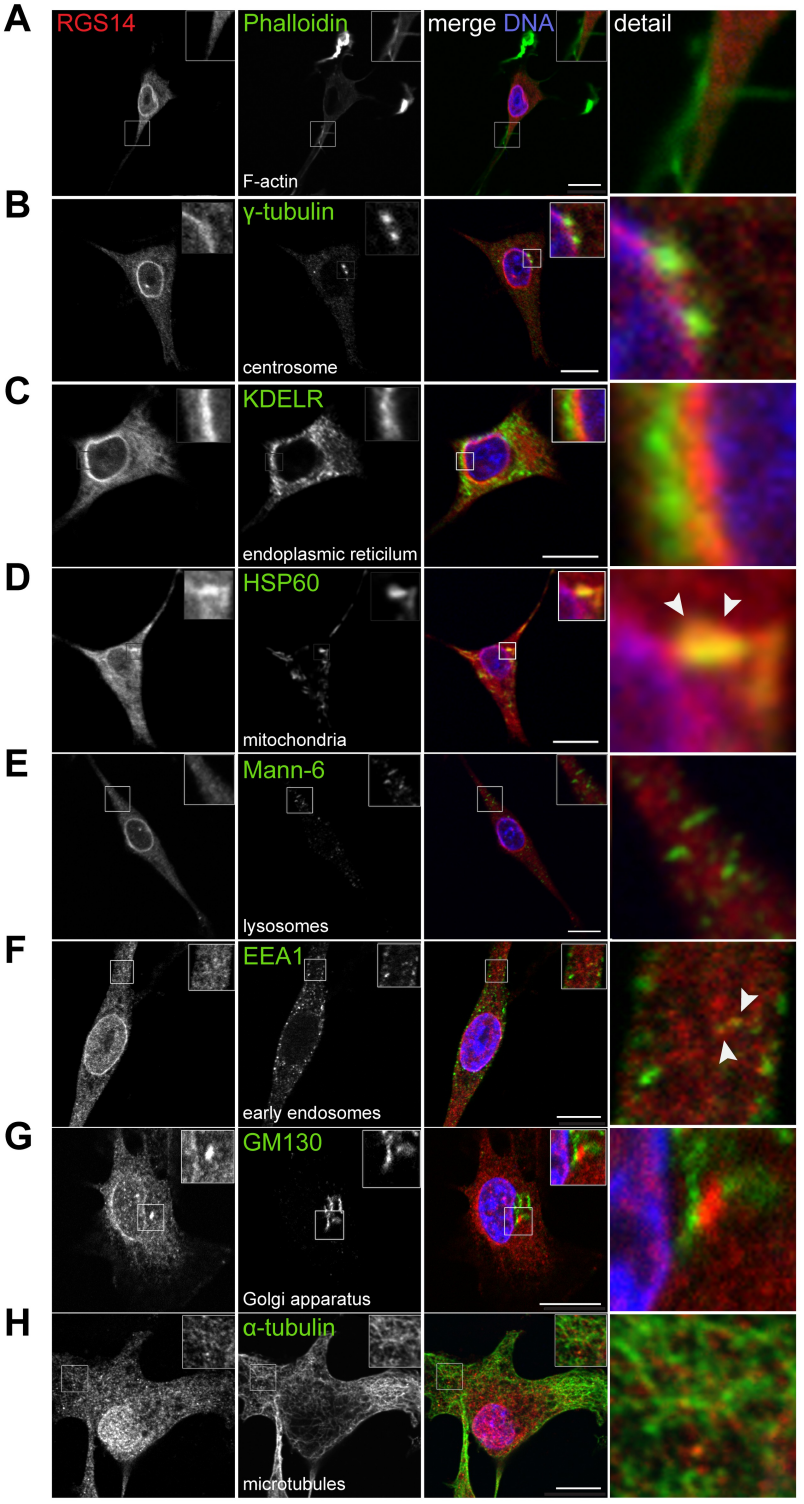
Endogenous RGS14 is enriched as puncta at various cytosolic compartments of B35 cells. B35 cells were fixed and co-stained with RGS14 polyclonal antibody (*red*) and one of several organelle markers (*green*): (A) Rhodamine phalloidin, F-actin; (B) γ-tubulin, centrosomes; (C) endoplasmic reticulum, KDELR; (D) HSP60, mitochondria; (E) lysosomes; (F) Mann-6; EEA1, early endosomes; (G) GM130, Golgi apparatus; (H) α-tubulin, microtubules. Nuclei were counterstained with Hoechst (*blue*). For B35 cells co-stained with rhodamine phalloidin to label F-actin (A) and antibody against mitochondrial marker HSP60 (D), cells were fixed with 4% paraformaldehyde in 1X PBS and permeabilized in 0.1% Triton-X. B35 cells co-stained with all other organelles were fixed with 4% PFA in PHEM cytoskeleton stabilizing buffer and permeabilized with methanol. Insets represent magnified boxed regions (2x magnification), and are enlarged to the right of the merged image (detail). Scale bar, 10 μm. White arrowheads point to regions of endogenous RGS14 colocalization with mitochondrial marker HSP60 (D) and early endosome marker EEA1 (F). Cells shown are representative of approximately 600 cells observed from 40 fields of view across 3 independent experiments.

To gain further insight into the cytosolic distribution of endogenous RGS14 outside of the nucleus, we co-stained B35 cells with antibodies against RGS14 and various endogenous organelle markers (Fig 3). Different fixation and permeabilization conditions were also evaluated for co-staining experiments because protein components of membranous organelles and cytoskeletal structures are preserved to varying extents by different immunofluorescence protocols [23]. Colocalization of RGS14 with organelle markers was evaluated visually by merging the red and green fluorescence channels (Fig 3). Using confocal microscopy, we found that endogenous RGS14 puncta are dispersed throughout the cytoplasm and along mictrotubules (Fig 3H). We did not find significant overlap between RGS14 puncta and organelle markers for filamentous actin (phallodin) (Fig 3A), centrosomes (γ-tubulin) (Fig 3B), the endoplasmic reticulum (KDEL receptor) (Fig 3C), lysosomes (Mann-6) (Fig 3E), or Golgi apparatus (GM130) (Fig 3G). We did, however, find partial colocalization between endogenous RGS14 and mitochondrial marker HSP60 (Fig 3D) and early endosome marker EEA1 (Fig 3F). In addition, within the cytoplasm, RGS14 puncta localized to a perinuclear region proximal to the centrosome that was maintained during various stages of the cell cycle (S4 Fig). Though not overlapping with the ER marker (Fig 3C), RGS14 staining was enriched in the juxtanuclear region in close proximity to the ER, along the nuclear periphery. In summary, endogenous RGS14 is localized within the cytoplasm as diffuse puncta that may include discrete endosomes adjacent to cytoskeletal filaments, and is notably concentrated at the juxtanuclear membrane adjacent to the ER.

### Endogenous RGS14 localizes to both the cytoplasmic and nuclear side of the nuclear envelope

**Fig 4:**
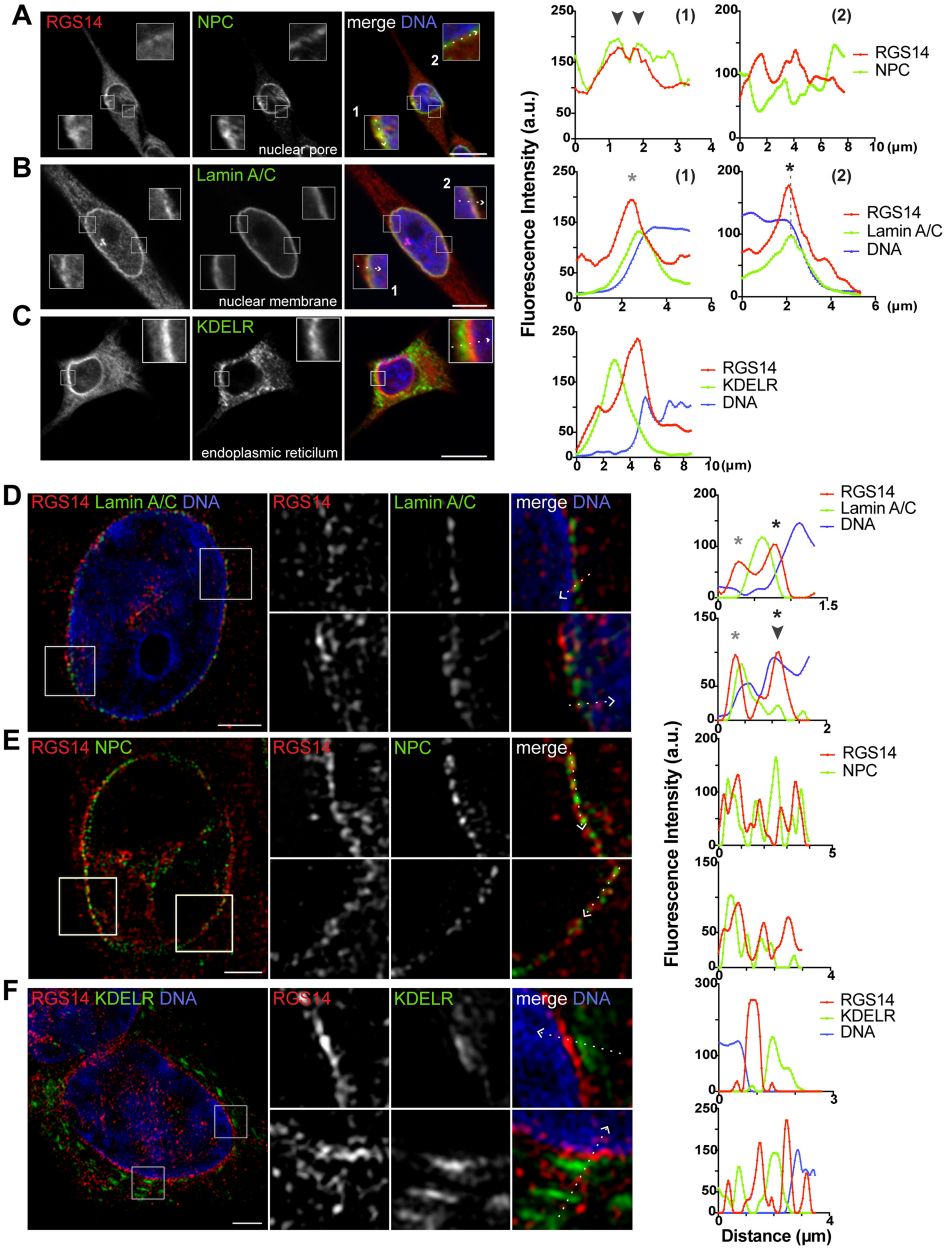
RGS14 localizes to both the cytoplasmic and nuclear sides of the nuclear envelope in B35 cells. B35 cells were immunostained for endogenous RGS14 (*red*) and nuclear pore complex proteins (NPC), nuclear membrane (Lamin A/C), or endoplasmic reticulum (KDELR) shown in *green*. Cells were counterstained with Hoechst to show DNA (*blue*). Optical mid sections were obtained by confocal microscopy (A-C; scale bar, 10 μm) or 3D-SIM (D-F; scale bar, 2 μm). (A-C) Insets in confocal images represent magnified boxed regions (2x magnification). (D-F) Boxed regions in 3D-SIM images are shown to the right at a 2X magnification. Graphs show fluorescence intensity (arbitrary units; a.u.) for each channel across the dotted white lines in the direction of the arrow in the merged images. RGS14 and nuclear pore complex proteins mainly localize in alternating puncta along the nuclear periphery (A, E). RGS14 localizes to both the cytoplasmic (*gray asterisk*) and nuclear (*black asterisk*) sides of the nuclear membrane (B, D), and between the endoplasmic reticulum and nucleus (C, F). Dotted gray line in graph 2 in B traces peak Lamin A/C fluorescence intensity to highlight that peak RGS14 fluorescence resides on the nuclear side of the nuclear membrane. Arrow heads indicate regions of apparent colocalization between RGS14 and the nuclear pore complex by confocal microscopy (A) or nuclear membrane by 3D-SIM (D). Cells shown are representative of approximately 600 cells observed from 40 fields of view across 3 independent experiments for confocal images and 50–75 cells for 3D-SIM images. Note that the same set of coverslips used to obtain confocal images were also used to obtain 3D-SIM images.

Due to the prominent localization and enrichment of endogenous RGS14 around the nuclear periphery and within the nucleus, we turned our focus to understanding in greater detail the localization of endogenous RGS14 within these subcellular regions. B35 cells were co-stained with antibodies against RGS14 and markers for the nuclear membrane (Lamin A/C), nuclear pore complex (monoclonal antibody 414, which labels FG-repeat containing nuclear pore complex proteins), and endoplasmic reticulum (KDELR) (Fig 4). Using confocal microscopy and 3D-SIM, fluorescence intensity analysis of lines drawn along the nuclear border revealed predominantly alternating patterns of RGS14 and nuclear pore complex (NPC) peak fluorescence signal, with some instances of apparent colocalization that could represent shuttling of endogenous RGS14 between the cytoplasm and nucleus through nuclear pores (Fig 4A and 4E). In addition, co-labeling B35 cells with an antibody against a marker for the nuclear membrane (Lamin A/C), showed RGS14 signal on both the cytoplasmic and nuclear side of the nuclear envelope (Fig 4B and 4D). Using 3D-SIM (Fig 4D–4F), we also observed some instances of colocalization between intranuclear Lamin A/C puncta and RGS14 (Fig 4D). RGS14 staining around the nuclear periphery consistently resided between the Hoechst-labeled DNA and ER with both confocal microscopy and 3D-SIM (Fig 4C and 4F).

### Endogenous RGS14 localizes to various subnuclear compartments associated with DNA-poor intranuclear channels and DNA-rich chromocenters

**Fig 5:**
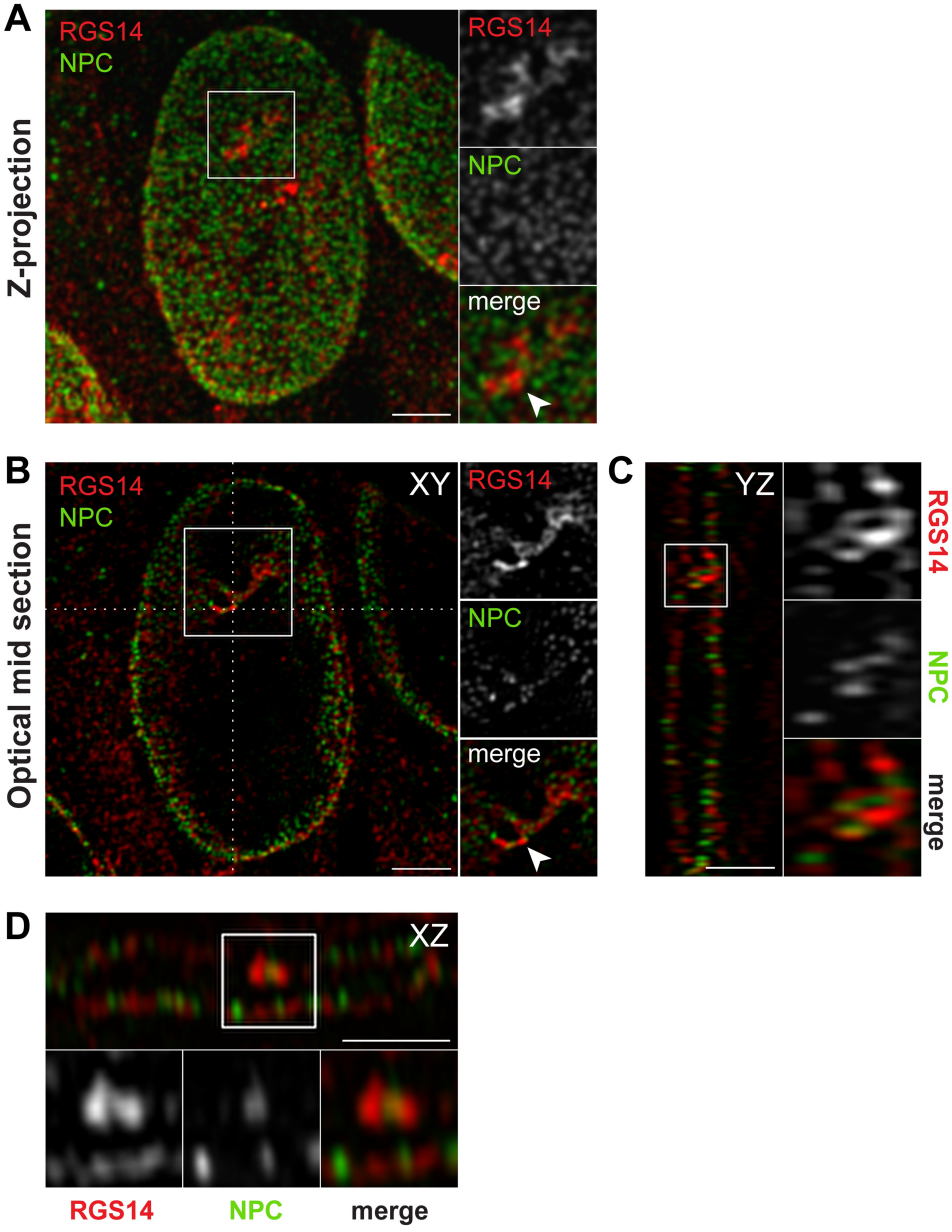
Endogenous RGS14 localizes to intranuclear channels enriched with nuclear pore complexes (NPC). Z-projection (A) and optical mid section (B) obtained with 3D-SIM of a B35 cell nucleus immunostained for endogenous RGS14 (*red*) and nuclear pore complex proteins (NPC; *green*). (C, D) Orthogonal views marked by the dotted white lines in B (XY-projection). White arrowheads in A and B point to the location of a DNA-poor intranuclear channel enriched with RGS14 and nuclear pore complex proteins (enlarged to the *right* or *bottom* of C and D, respectively). Note that RGS14 and nuclear pore complex proteins are localized to the same transnuclear channel, but do not co-localize. Cells shown are representative of approximately 75 cells observed across 3 independent experiments.

Serial sectioning with 3D-SIM of B35 cell nuclei further showed that clusters of RGS14 puncta formed tubule-like structures adjacent to NPCs in intranuclear bodies that were apparent when viewed in different optical planes (Fig 5A–5D). NPCs have previously been reported to reside in intranuclear channels in DNA-poor regions of the nucleus; NPC populated intranuclear channels also contain nuclear membrane proteins and are contiguous with the cytoplasm [24]. Though the precise function of NPC-rich intranuclear channels is ambiguous, evidence exists to support a role for these channels in facilitating nuclear import and export functions by reducing the distance between the cytoplasm and specific subnuclear compartment located deep within the nucleoplasm [25].

**Fig 6:**
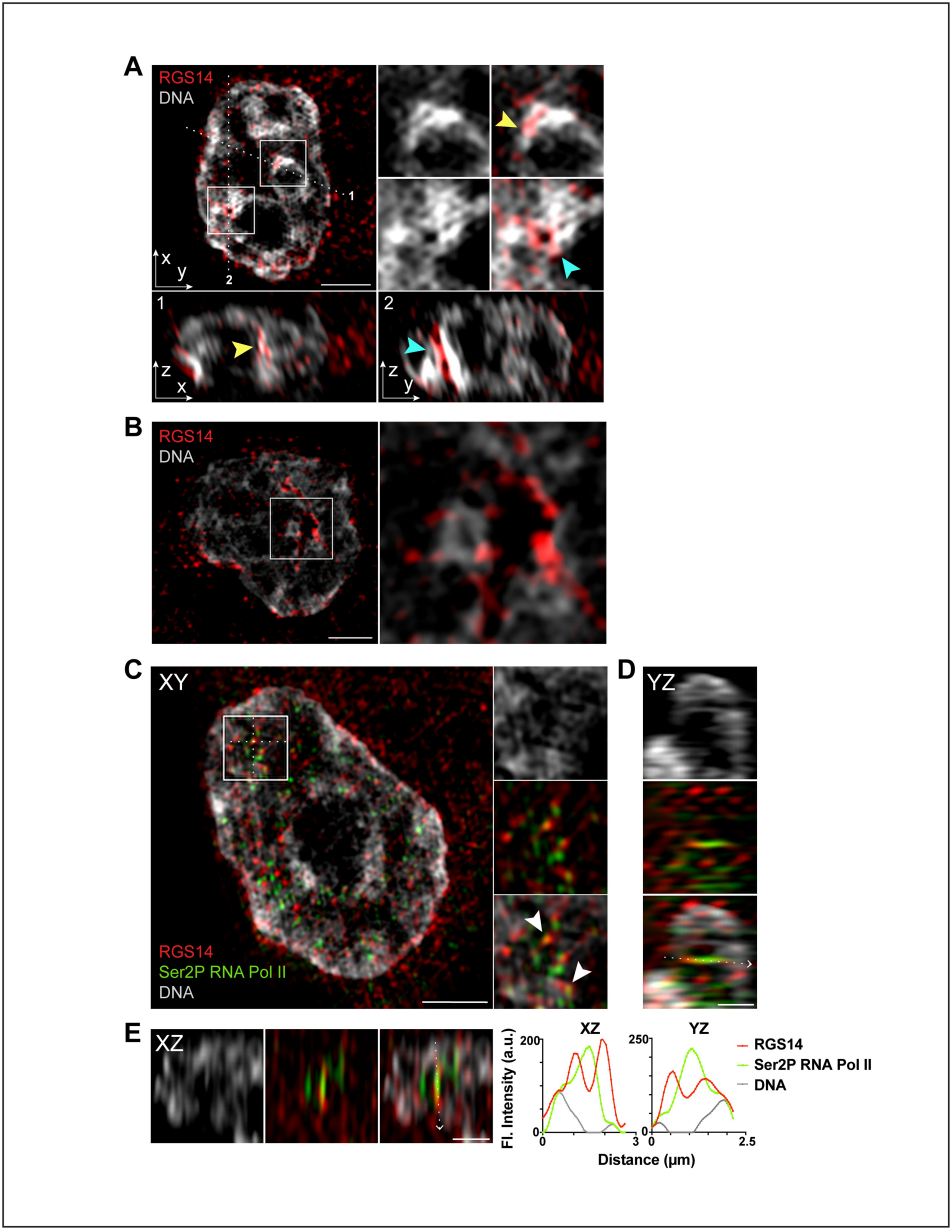
Endogenous RGS14 localizes to chromatin-rich compartments in close proximity with RNA polymerase II in the nucleus of B35 cells. Representative 3D-SIM images of the different types of RGS14 distributions observed in B35 cell nuclei. (A, B) B35 cell nuclei counterstained with Hoechst (*white/gray*) and immunostained with an anti-RGS14 polyclonal antibody (*red*). (A) Higher magnification images of boxed regions in the XY-projection (*top left*) are shown to the right. *Bottom row* shows orthogonal views marked by the dotted white lines in the XY-projection in the indicated planes (1, XZ; 2, YZ). Yellow arrowheads point to an area of enriched RGS14 staining located within a region of high intensity Hoechst-stained chromatin (chromocenter). Cyan arrows indicate enriched RGS14 staining within a DNA-poor intranuclear channel/tubule. Scale bar, 2 μm. (B) Optical mid section (XY-projection) of a B35 cell nucleus with RGS14 puncta enriched at the periphery of a DNA-rich chromocenter at a nuclear invagination. Higher magnification image of boxed region is shown to the right. Scale bar, 2 μm. (C) Optical mid section of a B35 cell nucleus counterstained with Hoechst (*gray*) and immunostained with an anti-RGS14 polyclonal antibody (*red*) and mAb H5 (*green*), which recognizes the 3′ end of active RNA Polymerase II (Ser2P RNA Pol II). Higher magnification image of boxed region is shown to the right. A subpopulation of RGS14 nuclear puncta colocalize with active RNA Polymerase II (Ser2P RNA Pol II) foci (white arrowheads). Scale bar, 2 μm. Orthogonal views YZ (D) and XZ (E) show RGS14 immunostaining wrapping around Ser2P RNA Pol II foci and spanning across a DNA-poor interchromatin compartment to connect two DNA-rich chromocenters. Scale bar, 1 μm. Graphs show fluorescence intensity (arbitrary units; a.u.) for each channel across the dotted white lines in the direction of the arrow in D and E. Cells shown are representative of approximately 75 cells observed across 3 independent experiments.

We further examined RGS14 clustering within the nucleus (Fig 6). 3D-SIM images of B35 cell nuclei stained for endogenous RGS14 and counterstained with Hoechst to visualize DNA revealed that RGS14 was enriched in both DNA-poor intranuclear channels that appeared to transect the nucleus and regions of densely Hoechst-stained chromatin (Fig 6A). In addition to their localization in DNA-poor intracnuclear channels, NPC proteins and lamins have also been reported to directly interact with condensed, transcriptionally inactive chromatin both at the nuclear periphery and deep within the nucleoplasm [26–29]. In this regard, we also found discrete RGS14 foci at the periphery of chromatin-rich (perichromatin) regions and along nuclear invaginations within the nucleus of some cells (Fig 6B).

Since the perichromatin RGS14 puncta localization pattern was consistent with the previously described localization of RNA polymerase II [30], we co-stained B35 cells with antibodies against RGS14 and an antibody that recognizes the active, elongating form of RNA polymerase II at the 3’ terminus (Ser2P-RNA Pol II) [30–33]. 3D-SIM images in different optical planes revealed a partial overlap and close proximity between perichromatin RGS14 puncta and Ser2P-RNA Pol II (Fig 6C–6E), suggesting that a subpopulation of RGS14 within the nucleus may be involved in transcriptional regulation.

### The cellular distribution of endogenous RGS14 is cell cycle-dependent in B35 cells

**Fig 7:**
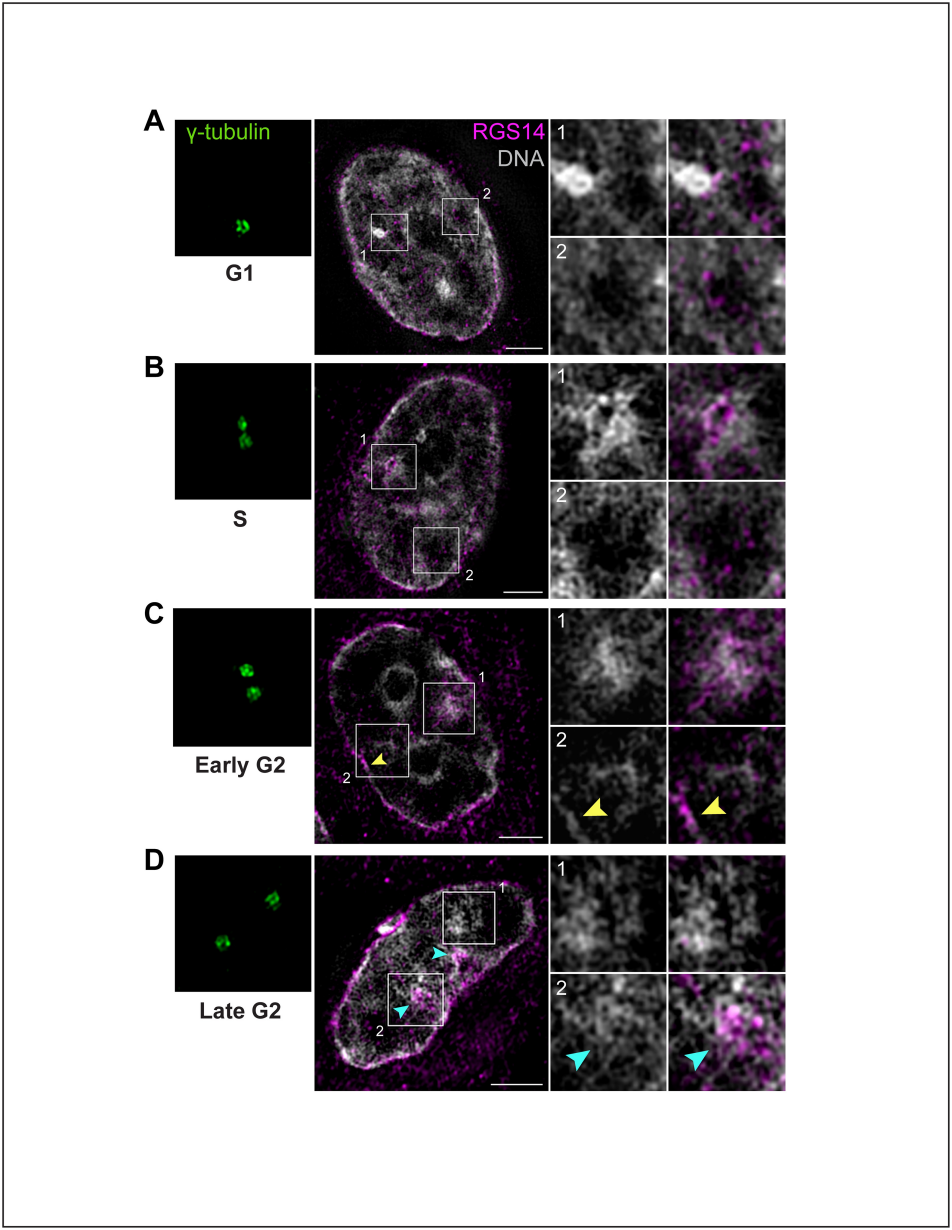
The distribution of endogenous RGS14 in the nucleus is cell cycle-dependent. (A-D) Representative 3D-SIM images of B35 cell nuclei synchronized at different stages of the cell cycle. Cells were synchronized at G1/S using a double thymidine block, fixed at different time points following release (as described in *Material and Methods*), and immunostained for endogenous RGS14 (*magenta*) and centrosome marker γ-tubulin (*green*), to confirm cell cycle stag, and counterstained with Hoechst (*gray*). Scale bar, 2 μm. In some cases, centrosomes did not lie in the same XY-plane as optimal mid sections through the nuclei and are thus shown in boxes to the left of each corresponding nucleus. Boxed regions are magnified and shown in numbered panels to the right. Representative images in G1, showing RGS14 puncta in the nucleus clustered along the periphery of regions of high intensity Hoechst-stained chromatin (chromocenter; A1) and are largely absent in dark DNA-poor interchromatin compartments (A2). In S and early G2, RGS14 puncta mainly localize within DNA-rich chromocenters (B1, C1) and along peripheral heterochromatin (C2, *yellow arrow*), and remain absent in DNA-poor interchromatin compartments (B2, C2). Representative images showing the disappearance of RGS14 puncta within medium-intensity chromatin territories (D1) during late G2/M as cells condense their nuclear genome while progressing into mitosis; instead, RGS14 puncta are concentrated within the two most highly compact chromocenters (D2, *cyan arrows*). Cells shown for each stage are representative of approximately 50–75 cells observed across 3 independent experiments.

Within a given population of B35 cells that were asynchronous in their stage of the cell cycle, we observed some variation in the distribution of RGS14 within the nucleus, suggesting that the localization of endogenous RGS14 in B35 cells may change as cells progress through the cell cycle. To examine this in more detail, we determined endogenous RGS14 subnuclear distribution within the nucleus at different cell cycle stages (Fig 7). To do so, we used different protocols to synchronize B35 cells in different cell cycle phases including serum starvation to synchronize cells in G0/G1 and a double thymidine block and release protocol to synchronize cells at S, early G2, and G2/M (late G2) stages of the cell cycle as described in *Materials and methods*. Synchronization and cell cycle stage were confirmed by staining B35 cells with an antibody against γ-tubulin to assess centrosome duplication and positioning. At all stages of the cell cycle, RGS14 foci within the nucleus that were not localized to intranuclear channels were restricted to the periphery of or within regions of Hoechst-stained DNA, and were absent in adjacent DNA-poor interchromatin compartments (IC), which are thought to serve as a preferential compartment for RNA processing and transport [34] (Fig 7). In B35 cell nuclei synchronized at G1, endogenous RGS14 staining was punctate along perichromatin regions between DNA-rich and DNA-poor subcompartments and was absent in highly compact chromatin areas typically associated with transcriptionally silenced genes [35] (Fig 7A). In S and early G2 phases, discrete RGS14 foci localized to perichromatin regions as in G1; unlike in G1, however, RGS14 staining was enriched within heterochromatin-containing areas at the nuclear periphery and nucleoplasm during S and early G2 phases (Fig 7B and 7C). At late G2, clusters of RGS14 foci were predominantly found within the most compact, densely stained chromatin domains (Fig 7D).

Our previous studies and those of others have reported that recombinant RGS14 can localize to centrosomes along with its binding partner Gαi [12,13]. We examined if this was the case for endogenous RGS14 in B35 cells (S4 Fig). Though our initial investigations revealed no significant co-localization between RGS14 and centrosome marker γ-tubulin in asynchronous B35 cells (Fig 3B), cell cycle synchronization experiments revealed that RGS14 localizes to regions near, but not directly associated with, centrosomes throughout the cell cycle (S4 Fig). Notably, the endogenous RGS14 signal in the vicinity of the centrosome is coincident with previously reported staining patterns of exogenous RGS14 when co-transfected with inactive Gαi1 [12,13] and may reflect an association of RGS14 with the microtubule organizing complex within the pericentriolar material.

### AlF_4_¯ -induced activation of endogenous G proteins alters the subcellular localization of endogenous RGS14 in B35 cells

**Fig 8:**
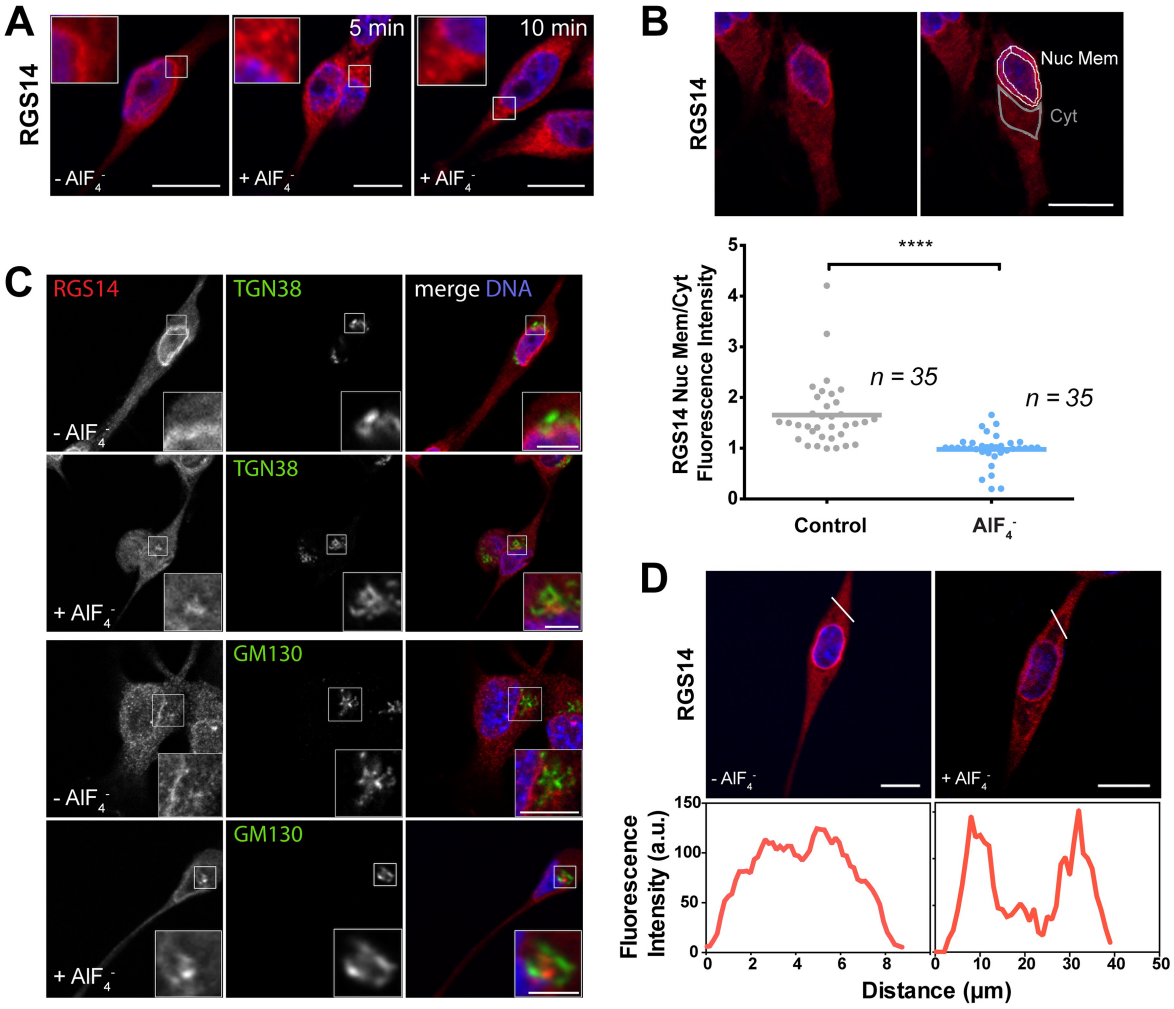
Activation of endogenous G proteins with AlF_4_¯ induces translocation of endogenous RGS14 from juxtanuclear membranes to cytosolic puncta and the plasma membrane. Confocal microscopy analysis (A, C, D) and quantification (B) of endogenous RGS14 translocation from the nuclear membrane after activation of endogenous G proteins with AlF_4_¯. A significant decrease in endogenous RGS14 localization around the nuclear membrane of B35 cells was observed 10 min after global G protein activation with AlF_4_¯. (A) Confocal images of B35 cells incubated with or without (control) AlF_4_¯ for indicated times and stained with an anti-RGS14 polyclonal antibody. Boxed regions are enlarged in the insets. (B) Representative confocal image of an untreated (control) B35 cell stained with the RGS14 polyclonal antibody (red) and counterstained with Hoechst DNA dye (blue) (left column). Right column shows the same cell with lines drawn around the nuclear membrane (white) and cytosol (gray) as described in *Materials and Methods*. Total fluorescence intensity was measured within the ring around the periphery of the nucleus (bounded by the white lines) and a comparable sized area within the cytosol (bounded by the gray lines) using ImageJ software. Scatterplot shows the ratio of nuclear membrane-to-cytosol localization of endogenous RGS14 in B35 cells following treatment with and without 10 min of AlF_4_¯ -induced G protein activation. Nuclear membrane-to-cytosol localization of RGS14 was determined by dividing the average fluorescence intensity (total fluorescence/area) around the nuclear membrane (Nuc Mem) by the average fluorescence intensity of a comparable area in the cytosol (Cyt) as described in *Materials and Methods*. Each point on the scatter plot represents the Nuc Mem/Cyt fluorescence intensity for a single cell immunostained with an RGS14 antibody and counterstained with Hoechst DNA dye to locate nuclei (n = 35 cells for each experimental condition, 3 independent experiments). Horizontal line shows mean Nuc Mem/Cyt intensity ratio. ****P<0.0001 (Student *t*-test). (C) Localization of endogenous RGS14 increased within clusters around the trans-Golgi network (anti-TGN38) and Golgi (anti- GM130) after G protein activation with AlF_4_¯ for 10 min, and (D) and at the plasma membrane in some cells after 15 min. Graphs (D) show fluorescence intensity (arbitrary units; a.u.) of RGS14 along the white lines in the above images. Scale bar, 10 μm in all cases.

The GPR motif on RGS14, which specifically binds to inactive Gαi1-GDP and Gαi3-GDP [13,36,37], is thought to be a key regulator of RGS14 cellular distribution in non-host cells (Shu et al., 2007). Our previous work showed that the RGS domain, which acts as a GAP for Gαi/o proteins [6–8], can also direct the cellular distribution of recombinant RGS14 in HeLa cells (Brown et al., 2015). Constitutive activation of ectopically expressed recombinant Gαi/o with aluminum tetrafluoride (AlF_4_¯), which activates G proteins by mimicking the transition state of GTP hydrolysis [38,39], is sufficient to recruit FLAG-RGS14 to the plasma membrane in HeLa cells [14]. Whether activation of endogenous G proteins affects the subcellular localization of endogenous RGS14 in its natural cellular environment, however, is unknown. Therefore, we examined whether activation of endogenous Gαi/o in B35 cells could recruit endogenous RGS14 to the plasma membrane in a similar manner. We treated B35 cells with AlF_4_¯ for various times, fixed and then processed them for immunofluorescence using the RGS14 polyclonal antibody (Fig 8). In untreated (control) cells, immunofluorescence and confocal imaging showed a relative enrichment of RGS14 around the nuclear membrane, as before. We observed that stimulation of cells with AlF_4_¯ lead to an increase in vesicle-like, punctate staining within the cytoplasm after 5 minutes and a notable decrease in nuclear membrane-to- cytoplasm fluorescence intensity by 10 minutes (Fig 8A). Quantifying this redistribution of RGS14 by examining a large number of cells, we observed a significant and very apparent redistribution of RGS14 from the nuclear periphery to the cytoplasm after 10 min with AlF_4_¯ (Fig 8B). These data indicate that constitutive G protein activation by AlF_4_¯ causes a redistribution of endogenous RGS14 from basal state cellular membranes around the nuclear membrane to vesicular structures within the cytoplasm and an accumulation of RGS14 puncta in a small juxtanuclear region. Co-staining cells with antibodies against RGS14 and specific markers for Trans-Golgi network (TGN38 protein) and cis-Golgi (GM130 protein), we found that the accumulation of RGS14 puncta at juxtanuclear regions following treatment with AlF_4_¯ were adjacent to these markers of the Golgi apparatus (Fig 8C). Notably, a subset of B35 cells showed a considerable increase in RGS14 localization at the plasma membrane after stimulation with AlF_4_¯ for 15 minutes (Fig 8D), whereas other cells continued to show an accumulation of RGS14 in vesicle-like structures within the cytoplasm, suggesting that AlF_4_¯- induced translocation of RGS14 is a dynamic process and RGS14 localization at the plasma membrane may be transient.

## Discussion

Understanding the spatiotemporal dynamics of proteins within host cells can provide vital insight into their native cellular functions. All previous information about the subcellular localization of RGS14 relied largely on exogenous expression of tagged recombinant protein in non-host cells. The mislocalization of exogenously expressed proteins is common and can give a false impression of the endogenous protein's distribution and *in vivo* functionalities [40,41]. Thus, the goal of these studies was to define, for the first time, a detailed assessment of the subcellular distribution and dynamic localization of endogenous RGS14 in a neuronal cell line and to compare and contrast the cellular behavior of endogenous versus recombinant RGS14. RGS14 is highly restricted in its protein distribution pattern, with protein expression limited to brain and certain lymphocytes [6]. Within rodent brain, RGS14 is expressed almost exclusively in adult CA2 hippocampal neurons [10,11] which, unfortunately, cannot be isolated for study. Therefore, to define endogenous RGS14 subcellular distribution, we utilized the only neuronal cell line reported to express endogenous RGS14, rat B35 neuroblastoma cells [6]. We found significant differences in the expression patterns and behavior of endogenous RGS14 in B35 cells compared with tagged recombinant RGS14 in B35 cells and other cell lines, in which RGS14 is not endogenously expressed. Whereas recombinant RGS14 is diffusely localized to the cytosol of various host cells, endogenous RGS14 exists as dispersed puncta within the cytoplasm, and is enriched along the nuclear periphery and localized on both sides of the nuclear envelope. Within the nucleus, RGS14 is observed within intranuclear channels adjacent to the nuclear pore complex, and is found as discrete puncta within both chromatin-rich and chromatin-depleted regions of the nucleus.

### RGS14 in the cytoplasm

We observe that endogenous RGS14 localizes in punctate structures along microtubules within the cytoplasm and partially colocalizes with mitochondria and early endosome markers. In CA2 hippocampal neurons, endogenous RGS14 is observed within pyramidal neurons at dendrites, spines, soma and axons [10], and we speculate that RGS14 is trafficked as a complex with it Gα partner attached to endosomes along microtubules and actin fibers. Consistent with this idea, RGS14 has previously been reported to bind microtubules and influence tubulin polymerization *in vitro* [42]. Thus, RGS14 may be involved in trafficking of endosomes along microtubules and/or actin filaments, or could be participating in G protein signaling from endosomes [43]. Germane to this idea, RGS14 contains a GPR motif and other GPR motif-containing proteins also have been reported to traffic to Golgi and endosomes [44], suggesting a role for the GPR-Gαi complex in regulating membrane trafficking events.

Though the colocalization of a subset of RGS14 puncta with mitochondria marker HSP60 was initially surprising as RGS14 does not contain a mitochondria localization signal, two studies have shown that endogenous Gαi1 localizes on the surface of mitochondria in HEK293T and HeLa cells [45,46]. The role of Gαi1 in relation to mitochondria is currently unknown, however, it is possible that the subpopulation of RGS14 puncta along mitochondria is interacting with Gαi1 to regulate some unknown mitochondrial function. In support of this, other G protein subunits have been reported to regulate mitochondria motility, morphology, and fusion [46,47]. In addition, possible interactions between Gαi1 and RGS14 in relation to microtubule and mitochondria dynamics may not be entirely separate. Though speculative, reported roles for RGS14 and Gαi1 in regulating tubulin polymerization [42,48] suggest that, in B35 cells, RGS14 could interact with Gαi to modulate tubulin-dependent mitochondria trafficking, anchorage, and/or fusion/fission initiation [49,50].

### RGS14 at the centrosome

Previous reports have shown that recombinant RGS14 can localize to centrosomes [12,13], along with its Gα binding partner Gαi1[51]. Our studies here indicate that a subpopulation of endogenous RGS14 is found surrounding centrosomes, though its role there is unclear. Based on these findings and previous findings showing recombinant RGS14 at centrosomes in other cell lines, we speculate that endogenous RGS14 in B35 cells may be operating within the pericentriolar matrix as part of the microtubule organizing center. Of note, other GRP motif-containing proteins have been shown to play a key role regulating microtubule pulling forces during asymmetric cell division [44]. Given that RGS14 is not expressed until postnatal day 7 in mice (Evans et al., 2014), it is unlikely that RGS14 plays a role centrosome-dependent neuron depolarization, migration, or axon formation, which occur between embryonic days 11 and 18 in the developing mouse brain [52]. Though it is commonly thought that the centrosome essentially becomes inactive as neurons mature, the possibility exists that the centrosome may be re-activated to generate microtubules in mature neurons in response to particular stimuli or challenges [53]. Alternatively, RGS14 pericentrosomal localization may be restricted to diving cells, however, many “key” cell cycle regulators and centrosomal proteins in undifferentiated cells have been shown to serve various important functions in adult neurons, including synaptic plasticity [54].

### RGS14 at the nuclear periphery

Surprisingly, our data indicate that endogenous RGS14 is highly enriched at membranes adjacent to and surrounding the nuclear envelope in host B35 cells. Since the outer nuclear membrane is contiguous with the ER, we speculate that RGS14 in this region may represent a newly synthesized pool of RGS14 on the outer ER membrane adjacent to the nuclear envelope that is positioned for trafficking to various subcellular compartments, including the endomembrane system, pericentriolar space, and nuclear periphery from which it can rapidly be shuttled into the nucleus. Positioned at the outer nuclear membrane, RGS14 could be involved in modulating nuclear positioning and cell polarity or could be dynamically transported through nuclear pores into the nuclear matrix, where it serves a currently unknown function. Regarding RGS14 trafficking to the nucleus, we observed endogenous RGS14 within NPC-rich intranuclear channels and invaginations. Though the precise function of NPC-rich intranuclear channels is uncertain, these are regarded as a highly specialized membrane subdomain of the ER [26] with evidence to support a role for these channels in facilitating nuclear import and export by reducing the distance between the cytoplasm and specific subnuclear compartments located deep within the nucleoplasm [25]. Similar to intranuclear channels, nuclear invaginations are typically populated with NPCs and are thought to play a role in improving macromolecular transportation between the cytoplasm and regions deep within the nuclear interior [25,55]. The localization of endogenous RGS14 to these specialized domains of the nuclear envelope suggests an as yet undefined role for RGS14 in nuclear transport.

### RGS14 in the nucleus

Early reports describing the subcellular localization of RGS14 in HeLa cells found that, under basal conditions, GFP- tagged RGS14 is predominantly localized to the cytoplasm and, in some cells, observed as perinuclear dot structures [12,13]. In addition, these early investigations found that GFP-RGS14 accumulated in the nucleus after application of the nuclear export inhibitor leptomycin B and localized to subnuclear compartments following mild heat shock-induced cellular stress [12,13]. These findings suggest that exogenously expressed recombinant RGS14 is a nucleocytoplasmic protein that rapidly shuttles into and out of the nucleus to serve diverse cellular roles, though its specific nuclear functions are still unclear. We observe that endogenous RGS14 behaves similarly, with a subset of the total cellular protein found within the nucleus in resting cells, and a majority of total cellular RGS14 accumulates in the nucleus when nuclear export is blocked with LMB treatment. These results confirm that endogenous RGS14 is in fact a cytoplasmic-nuclear shuttling protein. The observed nuclear localization of endogenous RGS14 in B35 cells is unsurprising given that RGS14 contains at least three putative nuclear localization signals (NLS) and a nuclear export signal (NES). Though native RGS14 in CA2 neurons from fixed hippocampal slices has not been reported in the nucleus [10], nuclear localization of RGS14 in neurons could depend on specific signals, including synaptic stimulation or neuronal stress. One intriguing possibility is that, in CA2 neurons, excitatory synaptic stimulation triggers RGS14 to be transported by microtubule-motors from the synapse to the nucleus. In the nucleus, RGS14 may directly or indirectly serve as a transcriptional regulator, thereby, influencing long-lasting changes in gene expression and subsequent synapse formation.

To date, signaling roles for RGS14 within the nucleus are unknown, but of great interest. Within the nucleus, we observed instances of colocalization between RGS14 with intranuclear lamin A/C puncta, and RGS14 elsewhere within the nucleus in both chromatin-rich and chromatin-poor regions. Intranuclear lamin foci have been reported to reside within DNA-rich regions of the nucleus and possibly to be involved in transcriptional regulation and/or RNA splicing [26,56,57]; however, the precise function of these foci is still unclear [26]. Of note, other RGS proteins have also been reported to translocate from the cytosol to the nucleus, including RGS10 [58,59], RGS12 [60,61], RGS7 [62,63], RGS6 [64], and RGS9-2 [63], though specific signaling roles for RGS proteins within the nucleus remain entirely unexplored. Nevertheless, the colocalization between endogenous RGS14 and intranuclear lamin-containing puncta in B35 cells raises the possibility that RGS14 may play a role in the regulation of gene expression [24], possibly through non-canonical G-protein signaling mechanisms.

A previous study has shown that overexpression of recombinant RGS14 in heterologous cell line can alter gene transcription, providing evidence for a possible role of RGS14 in the direct or indirect regulation of gene transcription [12]. Consistent with this idea, we observed RGS14 puncta in chromatic-rich regions of the nucleus with non-condensed DNA. RNA polymerase II has been reported to be enriched within these nuclear regions [30], and we observed RGS14 in close proximity with some overlap with the active elongating form (phosphorylated at Ser 2) of RNA polymerase II [30–33]. These findings suggest for the first time that a subpopulation of RGS14 within the nucleus may have a direct role in transcriptional regulation. During the G2 phase of the cell cycle, clusters of RGS14 foci were observed within the most compact, densely stained chromatin domains. Recruitment of genes to these compact chromocenters is known to lead to their silencing, whereas loose chromatin in the perichromatin regions that expand into the interchromatin compartment (IC) contain genes that are transcriptionally active [35]. Though the precise function of endogenous RGS14 within these various subnuclear compartments is currently unknown, these findings provide further evidence that RGS14 may play a role in transcriptional regulation and/or gene silencing.

### Translocation following G protein activation

Previous studies defining the behavior of recombinant tagged (GFP or FLAG) RGS14 in non-host cells show that tagged RGS14 localizes to the cytosol, but that it also can be recruited to the plasma membrane by co-expressed G protein binding partners, either activated Gαi/o- AlF_4_¯ or inactive Gαi1-GDP [13,14]. Quite unexpectedly, we observed no endogenous RGS14 at the plasma membrane in resting B35 cells. However, endogenous RGS14 can be stimulated by AlF_4_¯ (presumably bound to constitutively active Gαi/o on ER and Golgi membranes) to traffic from juxtanuclear membranes to the cytosol as puncta (endosomes) that can be trafficked to the plasma membrane. These data suggest that global G protein activation with AlF_4_¯ stimulates the trafficking of endogenous RGS14 from the nuclear periphery to cytosolic endosomes, likely derived from the cytoplasmic face of the ER and Golgi, some of which are trafficked to the plasma membrane. We speculate that endogenous RGS14 is translated in the cytosol and then finds a newly acylated Gα binding partner tethered to the outer leaflet of ER/Golgi membranes. Consistent with this idea, the RGS14 binding partner Gαi3 is known to be present and enriched on the cytoplasmic face of both Golgi membranes and endosomes where is thought to play a role in regulating anterograde protein trafficking to the plasma membrane [44,65].

In summary, we report here for the first time a detailed assessment of the subcellular distribution and dynamic localization of endogenous RGS14 in B35 neuroblastoma cells. The key findings show that endogenous RGS14 localizes to subcellular compartments not previously recognized in studies of recombinant RGS14 in unnatural host cells. The two most surprising observations were that RGS14 is not enriched at the plasma membrane, and that it is found within chromatin-rich regions of the nucleus in close proximity with active RNA Pol-II. These findings challenge models that RGS14 acts exclusively as a regulator of conventional GPCR-G protein signaling events at the plasma membrane. While this remains possible, as a minor pool of RGS14 is trafficked to the plasma membrane following AlF_4_¯activation of RGS14 G protein binding partners, the majority of RGS14 is not at the plasma membrane and remains in close proximity to the nucleus and within the nucleus for unknown functions unrelated to conventional GPCR signaling. That RGS14 was observed adjacent to active RNA Pol-II strongly suggests a possible novel role in the regulation of gene expression, perhaps as an enhancer or repressor, or possibly a role in regulating mRNA splicing. Also unexpected was the observation that RGS14 is most highly enriched at juxtanuclear membranes and can move from this compartment either to endosomes and the plasma membrane, or to the nucleus. How RGS14 is synthesized, how it is targeted to these membranes, and trafficked throughout the cell all remain topics for future study. These findings highlight novel cellular roles for RGS14 distinct from the regulation of conventional GPCR-G protein signaling, in particular undefined roles for RGS14 in the nucleus, a topic for ongoing and future studies.

## Supporting information

S1 Fig RGS14 polyclonal antibody recognizes GFP-RGS14 transfected into B35 cells. Confocal image of a B35 cell expressing GFP-RGS14 immunostained with RGS14 polyclonal antibody followed by Alexa 594 secondary antibody (*red*). The merged image shows complete colocalization of the RGS14 antibody signal and intrinsic GFP fluorescence (*green*). Scale bar, 10 μm.
(TIF)

S2 Fig Comparative effects of standard fixation and permeabilization protocols on the localization of endogenous RGS14. Confocal images of B35 cells processed for immunocytochemistry using various standard fixation and permeabilization protocols and immunostained with RGS14 polyclonal antibody. (A) Confocal images of B35 cells fixed by dehydration with organic solvents, methanol or acetone (5 min, −20°C). (B) Images of B35 cells fixed by cross-linking with 4% paraformaldehyde (PFA) in either 1X PBS or cytoskeleton stabilizing PHEM buffer and permeabilized with 0.1% Triton-X (10 min), 0.02% saponin (continuously), or methanol (5 min, −20°C). Scale bar, 10 μm. Note the distribution of RGS14 around the nuclear periphery in all protocols except with saponin permeabilization, which does not permeabilized the nuclear envelope. Cells shown are representative of approximately 600 cells observed from 40 fields of view across three independent experiments.
(TIF)

S3 Fig Translocation and accumulation of GFP-RGS14 in the nucleus of B35 cells upon treatment with leptomycin B. B35 cells were transfected with 500 ng GFP-RGS14 as described in *Materials and methods*. Twenty-four hours post-transfection, B35 cells transiently expressing GFP-RGS14 were imaged at 37°C, 5% CO_2_ with the DeltaVision OMX Blaze system under the wide-field setting. Prior to imaging, transfection media was replaced with Tyrode's solution (140 mM NaCl, 5 mM KCl, 1 mM MgCl_2_, 1 mM CaCl_2_, 0.37 mM NaH_2_PO_4_, 24 mM NaHCO_3_, 10 mM HEPES, and 0.1% glucose, pH 7.4). Cells were imaged for 1 min before the addition of leptomycin B (LMB) at a final concentration of 20 nM. Z-stacks were acquired every 10 min for a total of 90 min using a 488 nm laser. Scale bar, 10 μm. Montage is representative of 3 independent experiments.
(TIF)

S4 Fig Effect of cell cycle phase on the localization of RGS14 near the centrosome or Golgi. Confocal images of B35 cells synchronized at G1 phase of the cell cycle (A), or G2/M phase (B), co-stained with RGS14 polyclonal antibody (*red*) and centrosome marker, γ-tubulin, or Golgi marker, GM130 (*green*). Scale bar, 10 μm. White arrowheads point to the pericentriolar position of RGS14 puncta proximal to the centrosome during G1 and G2/M and ‘Golgi ribbon’ during G1. Cells shown are representative of approximately 300–360 cells observed from 40 fields of view across 3 independent experiments.
(TIF)

## References

[1] Hepler JR, Gilman AG (1992) G proteins. Trends Biochem Sci 17: 383–387. doi: 10.1016/0968-0004(92)90005-T 1455506 10.1016/0968-0004(92)90005-t

[2] Hamm HE (1998) The many faces of G protein signaling. J Biol Chem 273: 669–672. doi: 10.1074/jbc.273.2.669 9422713 10.1074/jbc.273.2.669

[3] Hollinger S, Hepler JR (2002) Cellular regulation of RGS proteins: modulators and integrators of G protein signaling. Pharmacol Rev 54: 527–559. doi: 10.1124/pr.54.3.527 12223533 10.1124/pr.54.3.527

[4] Shu F, Ramineni S, Hepler JR (2010) RGS14 is a multifunctional scaffold that integrates G protein and Ras/Raf MAPkinase signalling pathways. Cell Signal 22: 366–376. doi: 10.1016/j.cellsig.2009.10.005 19878719 10.1016/j.cellsig.2009.10.005 PMC2795083

[5] Vellano CP, Brown NE, Blumer JB, Hepler JR (2013) Assembly and function of the regulator of G protein signaling 14 (RGS14)·H-Ras signaling complex in live cells are regulated by Gαi1 and Gαi-linked G protein-coupled receptors. J Biol Chem 288: 3620–3631. doi: 10.1074/jbc.M112.440057 23250758 10.1074/jbc.M112.440057 PMC3561580

[6] Hollinger S, Taylor JB, Goldman EH, Hepler JR (2001) RGS14 is a bifunctional regulator of Galphai/o activity that exists in multiple populations in brain. J Neurochem 79: 941–949. 11739605 10.1046/j.1471-4159.2001.00629.x

[7] Cho H, Kozasa T, Takekoshi K, De Gunzburg J, Kehrl JH (2000) RGS14, a GTPase-activating protein for Gialpha, attenuates Gialpha- and G13alpha-mediated signaling pathways. Mol Pharmacol 58: 569–576. 10953050 10.1124/mol.58.3.569

[8] Traver S, Bidot C, Spassky N, Baltauss T, De Tand M-F, et al (2000) RGS14 is a novel Rap effector that preferentially regulates the GTPase activity of Gαo. Biochem J 350: 19 doi: 10.1042/0264-6021:3500019 10926822 PMC1221220

[9] Traver S, Splingard A, Gaudriault G, De Gunzburg J (2004) The RGS (regulator of G-protein signalling) and GoLoco domains of RGS14 co-operate to regulate Gi-mediated signalling. Biochem J 379: 627–632. doi: 10.1042/BJ20031889 15112653 10.1042/BJ20031889 PMC1224135

[10] Lee SE, Simons SB, Heldt SA, Zhao M, Schroeder JP, et al (2010) RGS14 is a natural suppressor of both synaptic plasticity in CA2 neurons and hippocampal-based learning and memory. Proc Natl Acad Sci U S A 107: 16994–16998. doi: 10.1073/pnas.1005362107 20837545 10.1073/pnas.1005362107 PMC2947872

[11] Evans PR, Lee SE, Smith Y, Hepler JR (2014) Postnatal developmental expression of regulator of G protein signaling 14 (RGS14) in the mouse brain. J Comp Neurol 522: 186–203. doi: 10.1002/cne.23395 23817783 10.1002/cne.23395 PMC3883939

[12] Cho H, Kim D-U, Kehrl JH (2005) RGS14 is a centrosomal and nuclear cytoplasmic shuttling protein that traffics to promyelocytic leukemia nuclear bodies following heat shock. J Biol Chem 280: 805–814. doi: 10.1074/jbc.M408163200 15520006 10.1074/jbc.M408163200

[13] Shu F, Ramineni S, Amyot W, Hepler JR (2007) Selective interactions between Gi alpha1 and Gi alpha3 and the GoLoco/GPR domain of RGS14 influence its dynamic subcellular localization. Cell Signal 19: 163–176. doi: 10.1016/j.cellsig.2006.06.002 16870394 10.1016/j.cellsig.2006.06.002

[14] Brown NE, Goswami D, Branch MR, Ramineni S, Ortlund EA, et al (2015) Integration of G protein α (Gα) signaling by the regulator of G protein signaling 14 (RGS14). J Biol Chem 290: 9037–9049. doi: 10.1074/jbc.M114.634329 25666614 10.1074/jbc.M114.634329 PMC4423691

[15] Hollinger S, Ramineni S, Hepler JR (2003) Phosphorylation of RGS14 by protein kinase A potentiates its activity toward G alpha i. Biochemistry 42: 811–819. doi: 10.1021/bi026664y 12534294 10.1021/bi026664y

[16] Oner SS, Maher EM, Breton B, Bouvier M, Blumer JB (2010) Receptor-regulated interaction of activator of G-protein signaling-4 and Galphai. J Biol Chem 285: 20588–20594. doi: 10.1074/jbc.C109.088070 20452976 10.1074/jbc.C109.088070 PMC2898320

[17] Nishi K, Yoshida M, Fujiwara D, Nishikawa M, Horinouchi S, et al (1994) Leptomycin B targets a regulatory cascade of crm1, a fission yeast nuclear protein, involved in control of higher order chromosome structure and gene expression. J Biol Chem 269: 6320–6324. 8119981

[18] Harper JV (2004) Synchronization of cell populations in G_1_/S and G_2_/M phases of the cell cycle Cell Cycle Control. New Jersey: Humana Press pp. 157–166. doi: 10.1385/1-59259-857-9:157

[19] Schreiber E, Matthias P, Müller MM, Schaffner W (1989) Rapid detection of octamer binding proteins with “mini-extracts”, prepared from a small number of cells. Nucleic Acids Res 17: 6419 2771659 10.1093/nar/17.15.6419 PMC318318

[20] Vellano CP, Maher EM, Hepler JR, Blumer JB (2011) G protein-coupled receptors and resistance to inhibitors of cholinesterase-8A (Ric-8A) both regulate the regulator of g protein signaling 14 RGS14·Gαi1 complex in live cells. J Biol Chem 286: 38659–38669. doi: 10.1074/jbc.M111.274928 21880739 10.1074/jbc.M111.274928 PMC3207400

[21] Goldenthal KL, Hedman K, Chen JW, August JT, Willingham MC (1985) Postfixation detergent treatment for immunofluorescence suppresses localization of some integral membrane proteins. J Histochem Cytochem 33: 813–820. doi: 10.1177/33.8.3894499 3894499 10.1177/33.8.3894499

[22] Gustafsson MGL, Shao L, Carlton PM, Wang CJR, Golubovskaya IN, et al (2008) Three-dimensional resolution doubling in wide-field fluorescence microscopy by structured illumination. Biophys J 94: 4957–4970. doi: 10.1529/biophysj.107.120345 18326650 10.1529/biophysj.107.120345 PMC2397368

[23] Stadler C, Skogs M, Brismar H, Uhlén M, Lundberg E (2010) A single fixation protocol for proteome-wide immunofluorescence localization studies. J Proteomics 73: 1067–1078. doi: 10.1016/j.jprot.2009.10.012 19896565 10.1016/j.jprot.2009.10.012

[24] Fricker M, Hollinshead M, White N, Vaux D (1997) Interphase nuclei of many mammalian cell types contain deep, dynamic, tubular membrane-bound invaginations of the nuclear envelope. J Cell Biol 136: 531–544. 9024685 10.1083/jcb.136.3.531 PMC2134289

[25] Malhas A, Goulbourne C, Vaux DJ (2011) The nucleoplasmic reticulum: form and function. Trends Cell Biol 21: 362–373. doi: 10.1016/j.tcb.2011.03.008 21514163 10.1016/j.tcb.2011.03.008

[26] Broers JLV, Ramaekers FCS, Bonne G, Yaou RB, Hutchison CJ (2006) Nuclear lamins: laminopathies and their role in premature ageing. Physiol Rev 86: 967–1008. doi: 10.1152/physrev.00047.2005 16816143 10.1152/physrev.00047.2005

[27] Lund E, Collas P (2013) Nuclear lamins: making contacts with promoters. Nucleus 4: 424–430. doi: 10.4161/nucl.26865 24213377 10.4161/nucl.26865 PMC3925686

[28] Morchoisne-Bolhy S, Geoffroy M-C, Bouhlel IB, Alves A, Audugé N, et al (2015) Intranuclear dynamics of the Nup107-160 complex. Mol Biol Cell 26: 2343–2356. doi: 10.1091/mbc.E15-02-0060 25904327 10.1091/mbc.E15-02-0060 PMC4462950

[29] Hou C, Corces VG (2010) Nups take leave of the nuclear envelope to regulate transcription. Cell 140: 306–308. doi: 10.1016/j.cell.2010.01.036 20144754 10.1016/j.cell.2010.01.036 PMC3040115

[30] Markaki Y, Gunkel M, Schermelleh L, Beichmanis S, Neumann J, et al (2010) Functional nuclear organization of transcription and DNA replication: a topographical marriage between chromatin domains and the interchromatin compartment. Cold Spring Harb Symp Quant Biol 75: 475–492. doi: 10.1101/sqb.2010.75.042 21467142 10.1101/sqb.2010.75.042

[31] Eskiw CH, Rapp A, Carter DRF, Cook PR (2008) RNA polymerase II activity is located on the surface of protein-rich transcription factories. J Cell Sci 121: 1999–2007. doi: 10.1242/jcs.027250 18495842 10.1242/jcs.027250

[32] Ghamari A, van de Corput MPC, Thongjuea S, van Cappellen WA, van Ijcken W, et al (2013) In vivo live imaging of RNA polymerase II transcription factories in primary cells. Genes Dev 27: 767–777. doi: 10.1101/gad.216200.113 23592796 10.1101/gad.216200.113 PMC3639417

[33] Sutherland H, Bickmore WA (2009) Transcription factories: gene expression in unions? Nat Rev Genet 10: 457–466. doi: 10.1038/nrg2592 19506577 10.1038/nrg2592

[34] Cremer M, Markaki Y, Zunhammer A, Cremer C, Cremer T (2001) Chromatin in the Cell Nucleus: Higher-order Organisation In: John Wiley & Sons, Ltd, editor. eLS. Chichester, UK: John Wiley & Sons, Ltd doi: 10.1002/9780470015902.a0005768.pub2

[35] Cremer T, Cremer C (2001) Chromosome territories, nuclear architecture and gene regulation in mammalian cells. Nat Rev Genet 2: 292–301. doi: 10.1038/35066075 11283701 10.1038/35066075

[36] Mittal V, Linder ME (2004) The RGS14 GoLoco domain discriminates among Galphai isoforms. J Biol Chem 279: 46772–46778. doi: 10.1074/jbc.M407409200 15337739 10.1074/jbc.M407409200

[37] Kimple RJ, De Vries L, Tronchère H, Behe CI, Morris RA, et al (2001) RGS12 and RGS14 GoLoco motifs are G alpha(i) interaction sites with guanine nucleotide dissociation inhibitor Activity. J Biol Chem 276: 29275–29281. doi: 10.1074/jbc.M103208200 11387333 10.1074/jbc.M103208200

[38] Berman DM, Kozasa T, Gilman AG (1996) The GTPase-activating protein RGS4 stabilizes the transition state for nucleotide hydrolysis. J Biol Chem 271: 27209–27212. doi: 10.1074/jbc.271.44.27209 8910288 10.1074/jbc.271.44.27209

[39] Sprang SR (1997) G proteins, effectors and GAPs: structure and mechanism. Curr Opin Struct Biol 7: 849–856. 9434906 10.1016/s0959-440x(97)80157-1

[40] Stadler C, Rexhepaj E, Singan VR, Murphy RF, Pepperkok R, et al (2013) Immunofluorescence and fluorescent-protein tagging show high correlation for protein localization in mammalian cells. Nat Methods 10: 315–323. doi: 10.1038/nmeth.2377 23435261 10.1038/nmeth.2377

[41] Margolin W (2012) The price of tags in protein localization studies. J Bacteriol 194: 6369–6371. doi: 10.1128/JB.01640-12 22961859 10.1128/JB.01640-12 PMC3497479

[42] Martin-McCaffrey L, Willard FS, Pajak A, Dagnino L, Siderovski DP, et al (2005) RGS14 is a microtubule-associated protein. Cell Cycle 4: 953–960. doi: 10.4161/cc.4.7.1787 15917656 10.4161/cc.4.7.1787

[43] Tsvetanova NG, Irannejad R, von Zastrow M (2015) G protein-coupled receptor (GPCR) signaling via heterotrimeric G proteins from endosomes. J Biol Chem 290: 6689–6696. doi: 10.1074/jbc.R114.617951 25605726 10.1074/jbc.R114.617951 PMC4358092

[44] Hewavitharana T, Wedegaertner PB (2012) Non-canonical signaling and localizations of heterotrimeric G proteins. Cell Signal 24: 25–34. doi: 10.1016/j.cellsig.2011.08.014 21907280 10.1016/j.cellsig.2011.08.014 PMC3205251

[45] Lyssand JS, Bajjalieh SM (2007) The heterotrimeric [corrected] G protein subunit G alpha i is present on mitochondria. FEBS Lett 581: 5765–5768. doi: 10.1016/j.febslet.2007.11.044 18037379 10.1016/j.febslet.2007.11.044

[46] Zhang J, Liu W, Liu J, Xiao W, Liu L, et al (2010) G-protein β2 subunit interacts with mitofusin 1 to regulate mitochondrial fusion. Nat Commun 1: 101 doi: 10.1038/ncomms1099 20981029 10.1038/ncomms1099

[47] Andreeva AV, Kutuzov MA, Voyno-Yasenetskaya TA (2008) G alpha12 is targeted to the mitochondria and affects mitochondrial morphology and motility. FASEB J 22: 2821–2831. doi: 10.1096/fj.07-104224 18367648 10.1096/fj.07-104224 PMC2493459

[48] Roychowdhury S, Panda D, Wilson L, Rasenick MM (1999) G protein alpha subunits activate tubulin GTPase and modulate microtubule polymerization dynamics. J Biol Chem 274: 13485–13490. 10224115 10.1074/jbc.274.19.13485

[49] Jourdain I, Gachet Y, Hyams JS (2009) The dynamin related protein Dnm1 fragments mitochondria in a microtubule-dependent manner during the fission yeast cell cycle. Cell Motil Cytoskeleton 66: 509–523. doi: 10.1002/cm.20351 19373772 10.1002/cm.20351

[50] Liu X, Weaver D, Shirihai O, Hajnóczky G (2009) Mitochondrial “kiss-and-run”: interplay between mitochondrial motility and fusion-fission dynamics. EMBO J 28: 3074–3089. doi: 10.1038/emboj.2009.255 19745815 10.1038/emboj.2009.255 PMC2771091

[51] Cho H, Kehrl JH (2007) Localization of Gi alpha proteins in the centrosomes and at the midbody: implication for their role in cell division. J Cell Biol 178: 245–255. doi: 10.1083/jcb.200604114 17635935 10.1083/jcb.200604114 PMC2064444

[52] Lewis TL, Courchet J, Polleux F (2013) Cell biology in neuroscience: Cellular and molecular mechanisms underlying axon formation, growth, and branching. J Cell Biol 202: 837–848. doi: 10.1083/jcb.201305098 24043699 10.1083/jcb.201305098 PMC3776347

[53] Baas PW, Falnikar A (2012) Re-evaluation of the Neuronal Centrosome as a Generator of Microtubules for Axons and Dendrites In: Schatten H, editor. The Centrosome. Totowa, NJ: Humana Press pp. 309–326. doi: 10.1007/978-1-62703-035-9_18

[54] Frank CL, Tsai L-H (2009) Alternative functions of core cell cycle regulators in neuronal migration, neuronal maturation, and synaptic plasticity. Neuron 62: 312–326. doi: 10.1016/j.neuron.2009.03.029 19447088 10.1016/j.neuron.2009.03.029 PMC2757047

[55] Popken J, Graf A, Krebs S, Blum H, Schmid VJ, et al (2015) Remodeling of the Nuclear Envelope and Lamina during Bovine Preimplantation Development and Its Functional Implications. PLoS ONE 10: e0124619 doi: 10.1371/journal.pone.0124619 25932910 10.1371/journal.pone.0124619 PMC4416817

[56] Muralikrishna B, Dhawan J, Rangaraj N, Parnaik VK (2001) Distinct changes in intranuclear lamin A/C organization during myoblast differentiation. J Cell Sci 114: 4001–4011. 11739632 10.1242/jcs.114.22.4001

[57] Jagatheesan G, Thanumalayan S, Muralikrishna B, Rangaraj N, Karande AA, et al (1999) Colocalization of intranuclear lamin foci with RNA splicing factors. J Cell Sci 112 (Pt 24): 4651–4661. 10574713 10.1242/jcs.112.24.4651

[58] Lee J-K, Tansey MG (2015) Physiology of RGS10 in neurons and immune cells. Prog Mol Biol Transl Sci 133: 153–167. doi: 10.1016/bs.pmbts.2015.01.005 26123306 10.1016/bs.pmbts.2015.01.005

[59] Burgon PG, Lee WL, Nixon AB, Peralta EG, Casey PJ (2001) Phosphorylation and nuclear translocation of a regulator of G protein signaling (RGS10). J Biol Chem 276: 32828–32834. doi: 10.1074/jbc.M100960200 11443111 10.1074/jbc.M100960200

[60] Chatterjee TK, Fisher RA (2002) RGS12TS-S localizes at nuclear matrix-associated subnuclear structures and represses transcription: structural requirements for subnuclear targeting and transcriptional repression. Mol Cell Biol 22: 4334–4345. doi: 10.1128/MCB.22.12.4334-4345.2002 12024043 10.1128/MCB.22.12.4334-4345.2002 PMC133853

[61] Chatterjee TK, Fisher RA (2000) Novel alternative splicing and nuclear localization of human RGS12 gene products. J Biol Chem 275: 29660–29671. doi: 10.1074/jbc.M000330200 10869340 10.1074/jbc.M000330200

[62] Rose JJ, Taylor JB, Shi J, Cockett MI, Jones PG, et al (2000) RGS7 is palmitoylated and exists as biochemically distinct forms. J Neurochem 75: 2103–2112. doi: 10.1046/j.1471-4159.2000.0752103.x 11032900 10.1046/j.1471-4159.2000.0752103.x

[63] Witherow DS, Wang Q, Levay K, Cabrera JL, Chen J, et al (2000) Complexes of the G protein subunit gbeta 5 with the regulators of G protein signaling RGS7 and RGS9. Characterization in native tissues and in transfected cells. J Biol Chem 275: 24872–24880. doi: 10.1074/jbc.M001535200 10840031 10.1074/jbc.M001535200

[64] Chatterjee TK, Fisher RA (2003) Mild heat and proteotoxic stress promote unique subcellular trafficking and nucleolar accumulation of RGS6 and other RGS proteins. Role of the RGS domain in stress-induced trafficking of RGS proteins. J Biol Chem 278: 30272–30282. doi: 10.1074/jbc.M212688200 12761220 10.1074/jbc.M212688200

[65] Zhang P, Kofron CM, Mende U (2015) Heterotrimeric G protein-mediated signaling and its non-canonical regulation in the heart. Life Sci 129: 35–41. doi: 10.1016/j.lfs.2015.02.029 25818188 10.1016/j.lfs.2015.02.029 PMC4415990

