# Supplementary figures and images for: Endogenous RGS14 is a cytoplasmic-nuclear shuttling protein that localizes to juxtanuclear membranes and chromatin-rich regions of the nucleus
Source: PLoS One. 2017 Sep 21;12(9):e0184497. doi: 10.1371/journal.pone.0184497 (PMC5608220; doi:10.1371/journal.pone.0184497)

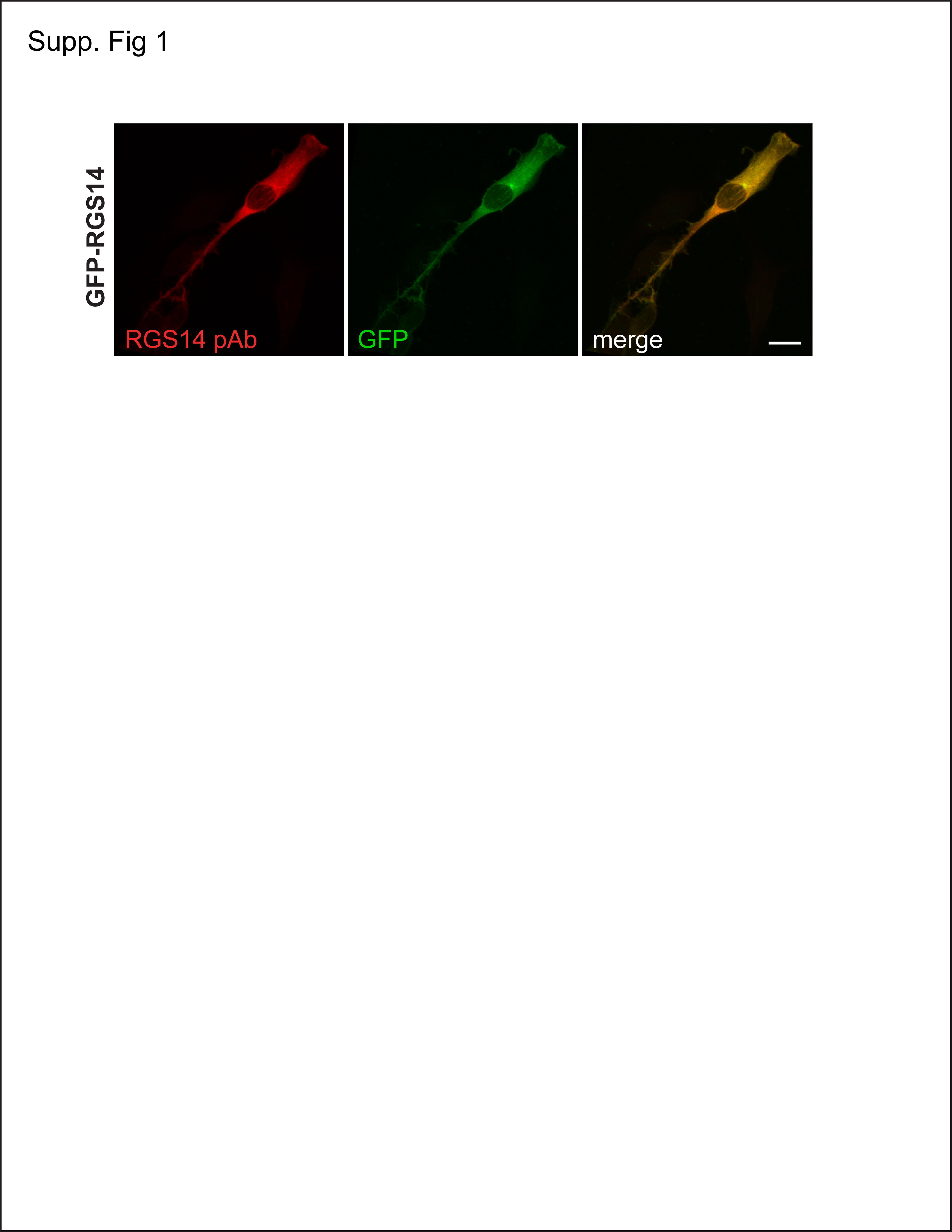

Supplement: S1 Fig — Confocal image of a B35 cell expressing GFP-RGS14 immunostained with RGS14 polyclonal antibody followed by Alexa 594 secondary antibody (red). The merged image shows complete colocalization of the RGS14 antibody signal and intrinsic GFP fluorescence (green). Scale bar, 10 μm. (TIF) [file pone.0184497.s001.tif]

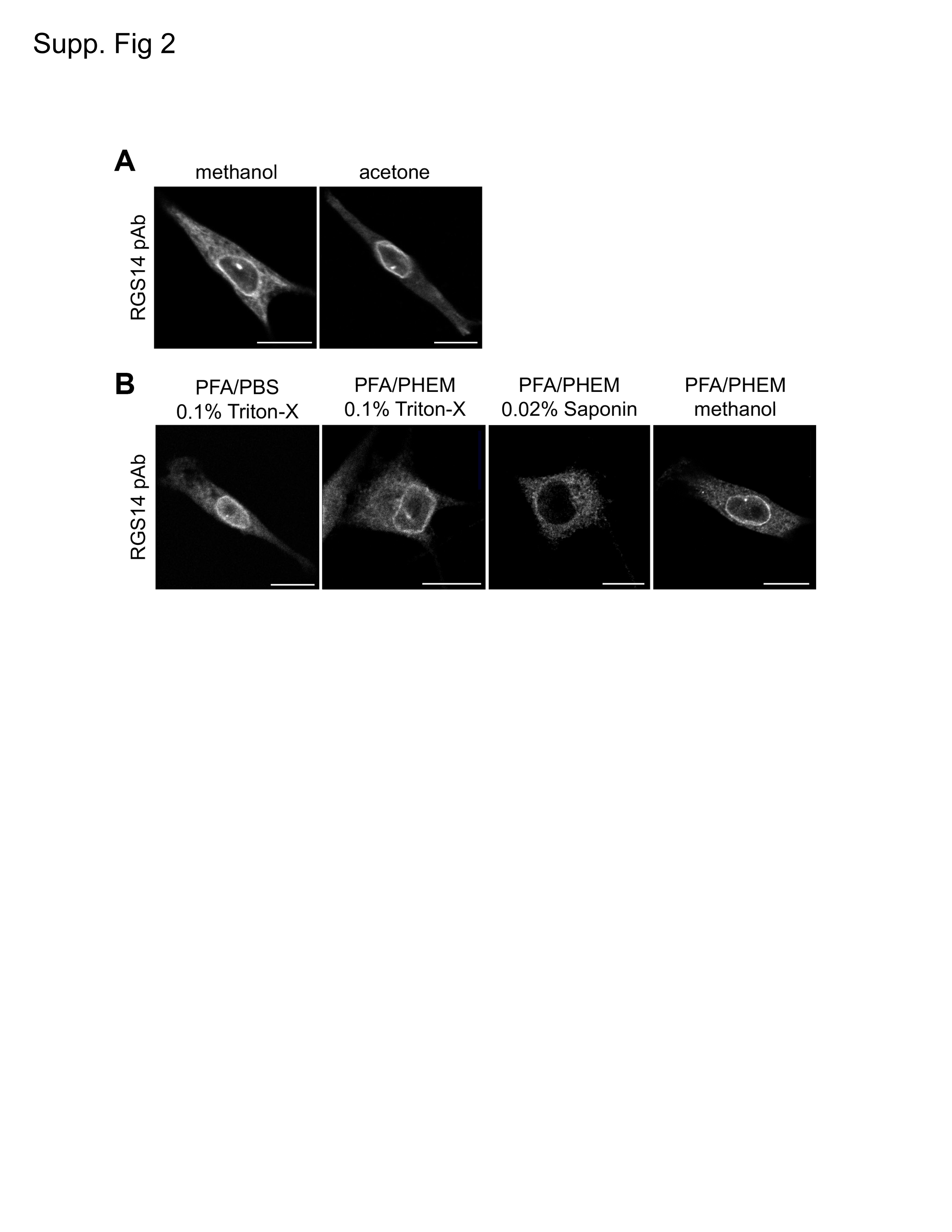

Supplement: S2 Fig — Confocal images of B35 cells processed for immunocytochemistry using various standard fixation and permeabilization protocols and immunostained with RGS14 polyclonal antibody. (A) Confocal images of B35 cells fixed by dehydration with organic solvents, methanol or acetone (5 min, −20°C). (B) Images of B35 cells fixed by cross-linking with 4% paraformaldehyde (PFA) in either 1X PBS or cytoskeleton stabilizing PHEM buffer and permeabilized with 0.1% Triton-X (10 min), 0.02% saponin (continuously), or methanol (5 min, −20°C). Scale bar, 10 μm. Note the distribution of RGS14 around the nuclear periphery in all protocols except with saponin permeabilization, which does not permeabilized the nuclear envelope. Cells shown are representative of approximately 600 cells observed from 40 fields of view across three independent experiments. (TIF) [file pone.0184497.s002.tif]

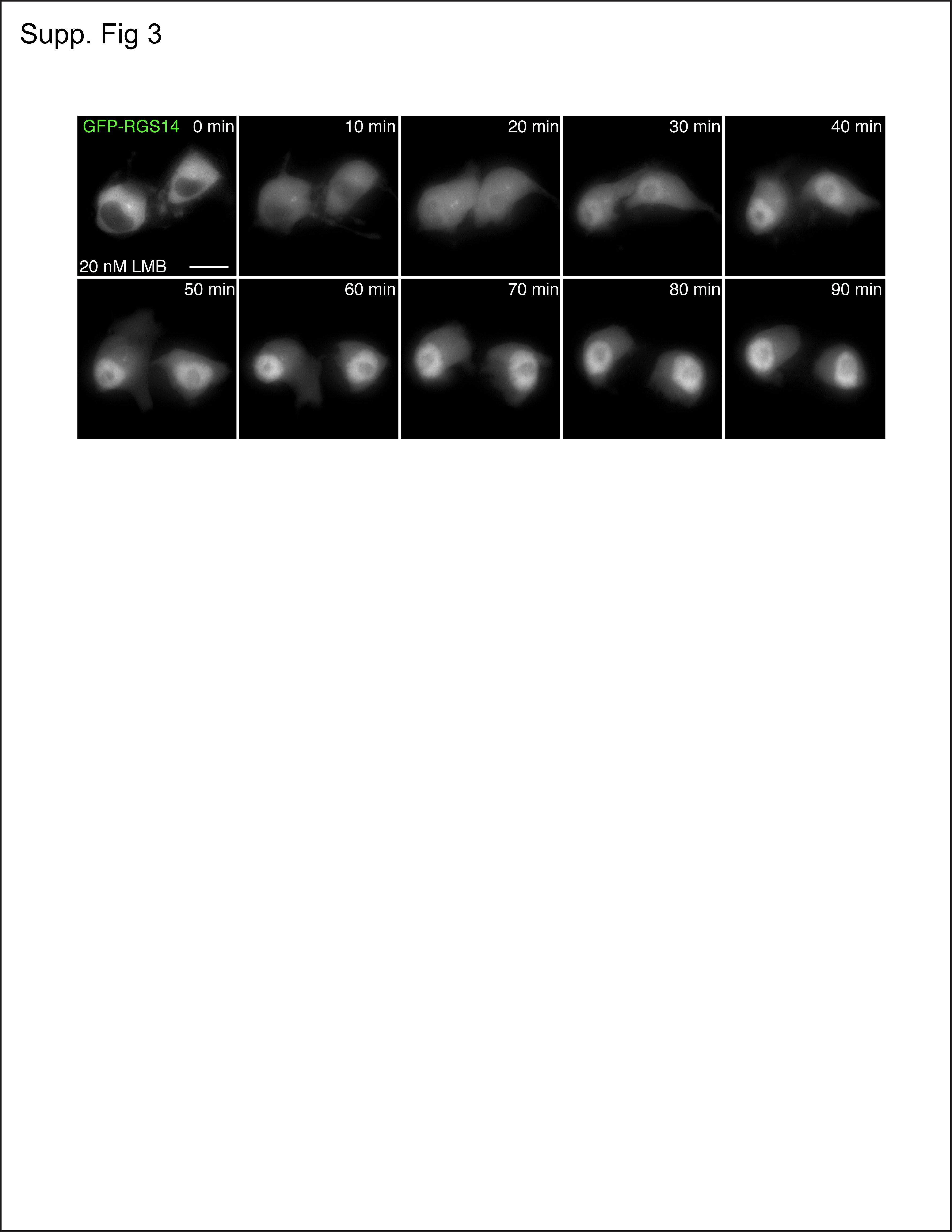

Supplement: S3 Fig — B35 cells were transfected with 500 ng GFP-RGS14 as described in Materials and methods. Twenty-four hours post-transfection, B35 cells transiently expressing GFP-RGS14 were imaged at 37°C, 5% CO2 with the DeltaVision OMX Blaze system under the wide-field setting. Prior to imaging, transfection media was replaced with Tyrode's solution (140 mM NaCl, 5 mM KCl, 1 mM MgCl2, 1 mM CaCl2, 0.37 mM NaH2PO4, 24 mM NaHCO3, 10 mM HEPES, and 0.1% glucose, pH 7.4). Cells were imaged for 1 min before the addition of leptomycin B (LMB) at a final concentration of 20 nM. Z-stacks were acquired every 10 min for a total of 90 min using a 488 nm laser. Scale bar, 10 μm. Montage is representative of 3 independent experiments. (TIF) [file pone.0184497.s003.tif]

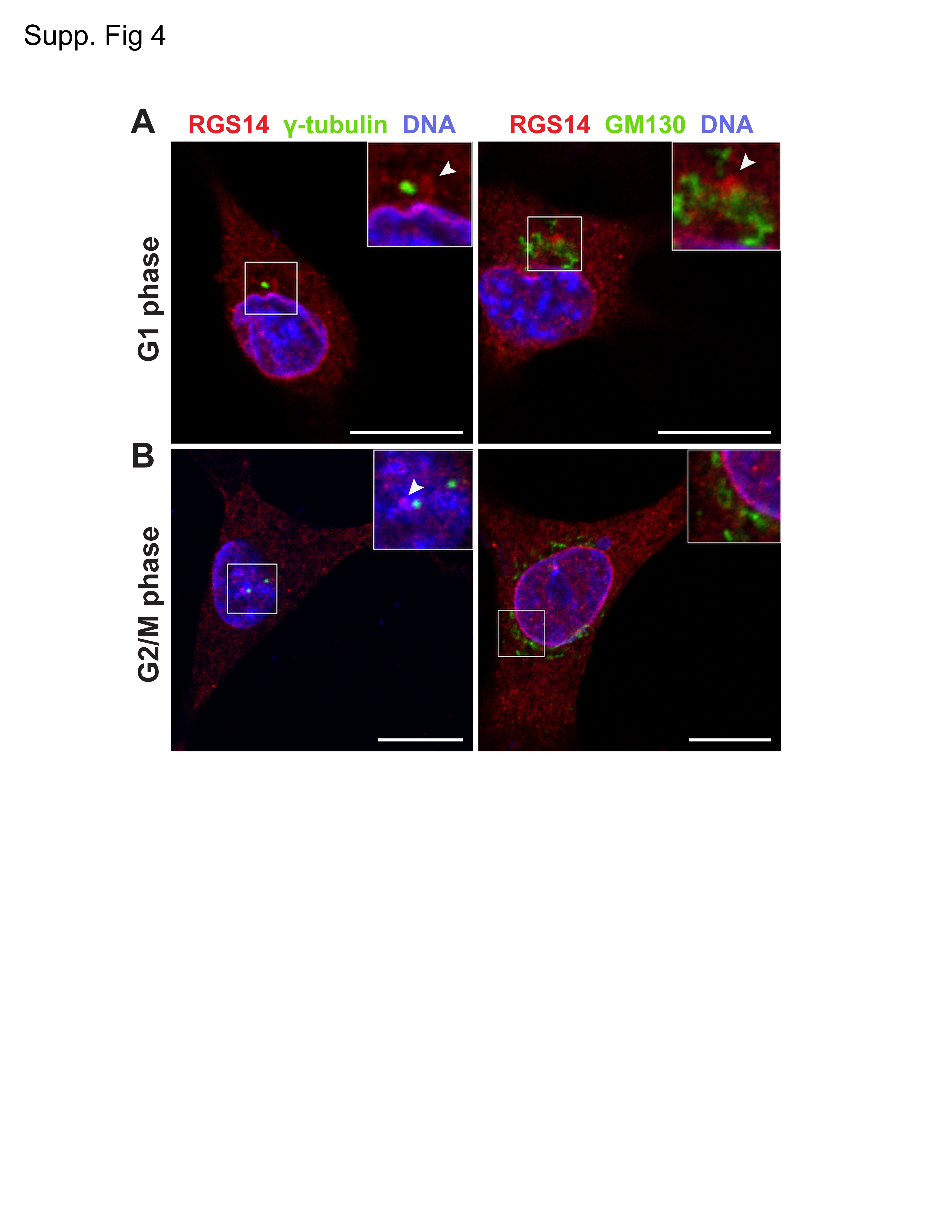

Supplement: S4 Fig — Confocal images of B35 cells synchronized at G1 phase of the cell cycle (A), or G2/M phase (B), co-stained with RGS14 polyclonal antibody (red) and centrosome marker, γ-tubulin, or Golgi marker, GM130 (green). Scale bar, 10 μm. White arrowheads point to the pericentriolar position of RGS14 puncta proximal to the centrosome during G1 and G2/M and ‘Golgi ribbon’ during G1. Cells shown are representative of approximately 300–360 cells observed from 40 fields of view across 3 independent experiments. (TIF) [file pone.0184497.s004.tif]
